# Global analysis of glycoproteins identifies markers of endotoxin tolerant monocytes and GPR84 as a modulator of TNFα expression

**DOI:** 10.1038/s41598-017-00828-y

**Published:** 2017-04-12

**Authors:** Mario M. Müller, Roland Lehmann, Tilman E. Klassert, Stella Reifenstein, Theresia Conrad, Christoph Moore, Anna Kuhn, Andrea Behnert, Reinhard Guthke, Dominik Driesch, Hortense Slevogt

**Affiliations:** 1 Septomics Research Center, Jena University Hospital, Jena, Germany; 2 BioControl Jena GmbH, Jena, Germany; 3 Leibnitz Institute for Natural Product Research and Infection Biology – Hans-Knöll-Institut, Jena, Germany; 4 Jena University Hospital, Integrated Research and Treatment Center - Center for Sepsis Control and Care (CSCC), Jena, Germany

## Abstract

Exposure of human monocytes to lipopolysaccharide (LPS) induces a temporary insensitivity to subsequent LPS challenges, a cellular state called endotoxin tolerance. In this study, we investigated the LPS-induced global glycoprotein expression changes of tolerant human monocytes and THP-1 cells to identify markers and glycoprotein targets capable to modulate the immunosuppressive state. Using hydrazide chemistry and LC-MS/MS analysis, we analyzed glycoprotein expression changes during a 48 h LPS time course. The cellular snapshots at different time points identified 1491 glycoproteins expressed by monocytes and THP-1 cells. Label-free quantitative analysis revealed transient or long-lasting LPS-induced expression changes of secreted or membrane-anchored glycoproteins derived from intracellular membrane coated organelles or from the plasma membrane. Monocytes and THP-1 cells demonstrated marked differences in glycoproteins differentially expressed in the tolerant state. Among the shared differentially expressed glycoproteins G protein-coupled receptor 84 (GPR84) was identified as being capable of modulating pro-inflammatory TNFα mRNA expression in the tolerant cell state when activated with its ligand Decanoic acid.

## Introduction

Glycoproteins are of particular importance for molecular and cellular recognition and for the modulation of intra- and intercellular crosstalk. Therefore, they are accounting for nearly 70% of pharmaceutical drug targets, e.g. G-protein-coupled receptors (GPCRs) and growth factor receptor tyrosine kinases and biomarkers^1, 2^. Mass spectrometry (MS)-based proteomic methods have emerged as powerful and universal tools to examine proteomes of individual cell types or whole organisms. However, glycosylated cell surface proteins and other membrane spanning proteins are often underrepresented in global proteomic analysis due to their low abundance and unfavorable biochemical properties e.g. the hydrophobicity of transmembrane domains and GPI-anchors^3, 4^. In recent years, several enrichment strategies for the targeted analysis of membrane proteins and transmembrane glycoproteins by MS were developed^5–8^ and affinity enrichment techniques targeting glycan chains on secreted and membrane anchored proteins using either hydrazide chemistry or lectins have been developed^9–11^. Proteomic identification and quantification of affinity enriched glycoproteins has been successfully used for the discovery of tissue-specific disease biomarkers in body fluids^12, 13^, or to analyze cellular states of differentiation^14–16^, and Bausch-Fluck *et al*.^17^ were recently able to obtain qualitative and quantitative information of the cellular N-glyco-surfaceome of 78 different cell lines that were distinguishable by the expression of specific cell surface proteins or on quantitative differences of particular N-glycoproteins. Therefore, cell surface glycoproteomics provides valuable information for cellular immunophenotyping by detecting and quantifying glycosylated cell surface marker proteins e.g. cluster of differentiation (CD) antigens or other cell surface associated glycoproteins. Whole cell glycoproteomics provides additional information, allowing comprehensive analysis of plasma membrane associated glycoproteins as well as glycosylated proteins from intracellular organelles that constitute around 50% of the cellular glycoproteome^10^.

Human monocytes are an essential component of the cellular immune system, playing a vital role in orchestrating innate immune responses to invading pathogens. They serve as the first line of host defense and are equipped with a variety of glycoprotein receptors to recognize and respond to infection by triggering inflammatory responses. As an early response to Gram-negative bacterial invasion, the detection of LPS by the toll-like receptor (TLR)4/CD14/MD2 complex is an example for the induction of monocytic cell activation in systemic infections. An important protective mechanism for the prevention of over-exuberant inflammation is endotoxin tolerance (ET). ET describes a phenomenon in which cells or organisms exposed to low concentrations of LPS enter a transient refractory state and do not respond adequately to further challenges with the same or other pathogen-associated molecular patterns (PAMPs)^18, 19^. The monocyte tolerant cell state is characterized by the secretion of high amounts of anti-inflammatory and low amounts of pro-inflammatory cytokines, higher phagocytic activity^20^, impaired antigen presentation^21^, and altered expression of molecules involved in tissue repair and matrix remodeling^22^. Changes in gene expression related to monocyte tolerance were confirmed in monocytes from patients during Gram-negative sepsis, acute coronary syndromes, cystic fibrosis and cancer^21, 23–28^. Epigenetic chromatin modifications at promotor regions^29^ and inhibitory changes in TLR-downstream signaling pathways^19, 27, 30^ contribute to monocyte reprogramming after initial activation. In addition, an altered expression of monocyte glycoproteins has been reported in the tolerant state. In monocytes from septic patients decreased expression of CD86 and MHC Class II (HLA-Dr) molecules, and increased secretion of IL1-RA was demonstrated that correlated with the state of tolerance and also with clinical outcome^20, 31^. As most cell surface expressed proteins are glycosylated, we hypothesized that a global analysis of glycoprotein expression changes elicited by LPS treatment is a useful approach to identify a characteristic glycoprotein signature indicative for the altered functional responses of tolerant monocytes. Furthermore, the identification of a characteristic glycoprotein expression pattern on monocytes and the identification of putative drug targets in the state of tolerance may have the potential to identify particular patient subgroups for the development of patient tailored immunomodulatory therapies in systemic infections.

In this study, we used purified peripheral blood CD14^+^ monocytes and the THP-1 monocytic cell line as a model system of monocyte functionality^32–35^. We used hydrazide-based solid-phase enrichment of whole cell derived glycosylated proteins^9^ after sodium dodecylsulfate (SDS)-mediated cell lysis for a comprehensive analysis of intra- and extracellular expressed glycoproteins in LPS-tolerized cells. We present a detailed glycoproteome data set of CD14^+^ monocytes and THP-1 cells, resulting in the identification and quantification of 1109 annotated glycoproteins from purified CD14^+^ monocytes and 1189 glycoproteins from THP-1 cells. In CD14^+^ monocytes and THP-1 cells 77 and 117 LPS-mediated transient or long-lasting glycoprotein expression changes were identified, respectively. Among the LPS regulated glycoproteins upregulated in both monocytes and THP-1 cells, we identified GPR84, a G protein-coupled receptor for medium-chain fatty acids. Activation of GPR84 with its specific ligand Decanoic acid (Capric acid) after pre-stimulation with LPS modulated TNFα mRNA production capability in endotoxin tolerant cells.

## Experimental Section

### Cell Culture and Stimulation

THP-1 cells (ATCC) were cultured in RPMI medium (Invitrogen, UK) supplemented with 10% fetal calf serum, Penicillin and Streptomycin (Invitrogen, UK). Human monocytes were isolated from buffy coats, obtained from healthy male donors (University Hospital Jena). Briefly, PBMCs were isolated by Ficoll (Ficoll-Paque Premium, GE Healthcare, UK) gradient density centrifugation in Leucosep^TM^ tubes and remaining erythrocytes were lysed with ACK lysing buffer (Gibco, US). Monocytes were enriched via MACS negative selection using Monocyte isolation Kit II (Miltenyi Biotec GmbH, Germany). MACS-purified monocytes were used for all stimulation experiments in RPMI medium. For glycoproteomics, the unstimulated or stimulated MACS-separated cell population was incubated for 24 h or 48 h and, subsequently, labelled with FITC-conjugated anti-CD14 antibodies (clone 61D3, eBioscience) and PE-coupled lineage markers: anti-CD3 (clone UCHT1, BD Biosciences), anti-CD19 (clone HIB19, BD Biosciences) and anti-CD42 (clone HIP1, Immunotools, Germany). CD14-positive cells were collected by gating on the FITC-positive and PE-negative cell population using a FACS-ARIA II (BD Biosciences) flow cytometer, achieving a CD14^+^ monocyte population of 97.5–98.3% purity. 1 × 10^7^ (glycoproteomics) or 4 × 10^6^ (qPCR) cells were either left untreated or stimulated with 50 ng/ml LPS (ultrapure LPS from *Salmonella minnesota* R95, InvivoGen) for the indicated times. Cells were collected by centrifugation and washed 6 times with PBS. Cells were re-suspended with 10 µl PBS and lysed in 200 µl 2% SDS in PBS. After heating at 95 °C for 5 min, samples were stored at −80 °C until further use. For tolerance induction and qPCR analysis cells were either left untreated or pre-stimulated with 50 ng/ml LPS for 24 h or 48 h. Two hours after LPS treatment, 500 µM Capric acid (Sigma-Aldrich) was added to some samples for 22 h. After 24 h of pre-stimulation, cells were washed and re-stimulated with 50 ng/ml LPS for two hours, collected by centrifugation, and the cell pellets were lysed in RLT buffer (Qiagen, Germany) and stored at −80 °C until further use.

### RT‑PCR and Quantitative PCR

To analyze gene expression of target genes, total RNA was isolated using the RNeasy kit from Qiagen (Qiagen, Germany). Residual genomic DNA was degraded by DNaseI (Qiagen, Germany). RNA concentration was measured with a NanoDrop D-1000 Spectrophotometer (Thermo-Fisher Scientific, Germany). Complementary DNA (cDNA) was synthesized from 2 μg of RNA using the High Capacity cDNA Reverse Transcription Kit (Applied Biosystems, UK) following the manufacturer’s instructions. PCR was carried out as described^36^. Briefly, PCR was done on a S1000™ Thermal Cycler (BioRad, UK) in a 25 μl reaction volume (0.2 μM primers, 1 U Taq DNA polymerase (5-Prime, UK) and 200 μM dNTPs). Thermal conditions included an initial 95 °C denaturation step for 3 min, and then 35 cycles of 10 s at 94 °C, 30 s at 60 °C and 30 s at 72 °C. PCR products were separated on agarose gels and visualized with Ethidium bromide under a UV-Transilluminator to confirm the expected amplicon size. A complete primer list can be found in Supplementary Table S1. To quantify relative gene expression, a Corbett Rotor-Gene 6000 (Qiagen, Germany) was used for real-time qPCR. Each sample was analyzed in duplicate in a total reaction volume of 20 μl containing 10 μl of 2 × SensiMix SYBR Master Mix (Bioline, UK) and 0.2 μM of each primer pair, assembled using the CAS-1200 pipetting robot (Qiagen, Germany). The cycling conditions were 95 °C for 10 min followed by 40 cycles of 95 °C (15 s), 60 °C (20 s) and 72 °C (20 s). RT-negative samples were included as controls. Specificity of the qPCRs was assessed by melting curve analysis. Relative expression of target genes was analyzed using a modified method described by Pfaffl *et al*.^37^. The stability of the housekeeping genes was assessed using the BestKeeper algorithm^38^.The normalized RQ (NRQ) values were log2-transformed for further statistical analysis with GraphPad PRISM v5.0. Significance was tested using 1-way ANOVA.

### Flow Cytometry

For detection of branched complex glycoconjugates, cells were stimulated with 50 ng/mL LPS for 24 h and 48 h hours or left untreated. Cells were washed in ice-cold 1% bovine serum/PBS and incubated with 2 µg/200,000 cells Alexa488-coupled PHA-L lectin (Invitrogen). For the detection of differentially expressed glycoproteins after LPS treatment, cells were stimulated for 4 h, 24 h, or 48 h with or without LPS and labelled with specific fluorescent-dye conjugated antibodies CD54-APC, Immunotools; CD14-FITC, CD169-APC, CD86-PerCP-Cy5 CD127-APC, CD274-APC, CLEC12A-PE, all eBiosciences; CD319-Alexa647, BD Biosciences; CD80-PE, Miltenyi Biotec). After washing, flow cytometry was performed with an Attune Acoustic Focusing Cytometer Attune (Applied Biosystems) or FACS-ARIA II (BD Biosciences). For visualization, FCS-files were loaded into FlowJo. Expression of glycoprotein marker proteins was analyzed by gating on live CD14^+^ cells.

### Glycoprotein Enrichment for Glycoproteomics

After thawing, 100 µl of lysate was treated with Benzonase nuclease (1 µl, Sigma-Aldrich) for 1 h at 37 °C and centrifuged at 24.000 × g for 20 min (20 °C). The supernatant was subjected to buffer exchange using polyacrylamide spin desalting columns (7 K MWCO, Pierce) equilibrated with 100 mM sodium acetate, pH 5.5. Samples were oxidized with 10 mM sodium (meta)periodate (Sigma-Aldrich) for 30 min at room temperature in the dark. Sodium periodate was removed by a second buffer exchange using polyacrylamide desalting spin columns equilibrated with PBS pH = 7.5. Oxidized sugar groups were immobilized on 50 µl (bead volume) Ultralink Hydrazide Gel (Pierce) overnight at room temperature with aniline (Sigma-Aldrich) as catalyst. Next day, non-glycoproteins were removed by washing the resin three times with the following buffers: 1% SDS/PBS, 8 M Urea in 100 mM Tris/HCl pH = 8.0, 1 M NaCl in 100 mM Tris/HCl pH = 8.0, 20% CH_3_CN/10 mM Tris/HCl pH = 8.0. After the second Urea wash, proteins were reduced by addition of 100 mM DTT in 8 M Urea Tris/HCl pH = 8.0 (60 min, room temperature) and alkylated with 50 mM Iodacetamid in 8 M Urea/100 mM Tris/HCl pH = 8.0 (30 min, room temperature in the dark). After washing, the resin was equilibrated with 50 mM NH_4_HCO_3_ and re-suspended in 30 µl 50 mM NH_4_HCO_3_ containing 2 µg sequencing grade trypsin (Pierce) and digested on-resin over night at 37 °C. Trypsin-released peptides were collected and dried. The resin was further extensively washed (as described above) and resuspended in 50 mM NH_4_HCO_3_ containing 0.5 U PNGase F. After enzymatic release of N-glycopeptides (37 °C, overnight) deglycosylated peptides were collected and dried.

### Mass Spectrometric Analysis

Samples were reconstituted in 0.1% formic acid and peptide concentrations were measured using a NanoDrop spectrometer. 2 µg of tryptic peptides and 0.5 µg of deglycosylated peptides were analyzed in each LC-MS/MS run in duplicates on an Orbitrap Fusion (Thermo Scientific) coupled to a Dionex Ultimate 3000 (Thermo Scientific) nanoelectrospray ion source. Samples were loaded on a 2 cm C18 trap column (Acclaim PepMap100, Thermo Scientific) and separated using a 2.5 h non-linear gradient (2–80% acetonitrile/0.1% formic acid, flow rate 300 nl/min) on a 50 cm C18 analytical column (75 µm PepMap RSLC, Thermo Scientific). Full MS scans were acquired with resolution 120.000 at m/z 400 in the Orbitrap analyzer (m/z range 400–1600, quadrupole isolation). MS1 parent ions were fragmented by higher energy collisional dissociation (HCD, 30% collision energy) and fragment ion spectra were acquired (in the order highest charge to least charge and least intense to highest intensity during a max. 4 sec cycle time) in the ion trap in rapid mode (m/z start 110). The following conditions were used: spray voltage of 2.0 kV, heated capillary temperature of 275 °C, S-lens RF level of 60%, ion selection threshold of 50,000 counts for HCD, maximum ion accumulation times of 50 ms (AGC 5 × 10^5^) for full scans and 35 ms (AGC 1 × 10^4^) for HCD.

### Protein Identification, Quantification and Statistics

All RAW files were searched against the human UniProt database (Version 05.2016, reviewed sequences) with MaxQuant version 1.5.5.1 (Max Planck Institute of Biochemistry, ref. 39. The parameters were set as follows: main search peptide tolerance: 4.5ppm; enzyme: trypsin, max. 2 missed cleavages; static modification: cysteine carbamidomethylation; variable modification in the tryptic peptide fraction: methionine oxidation; variable modification in PNGase F fractions: methionine oxidation and asparagine deamidation. PSM (peptide specific matches) and protein FDR was set to 0.01. For advanced identification the Second Peptide Search in MS2 spectra and the Match Between Runs feature were enabled. Label-free quantification of proteins with normalization was done in MaxQuant^40^. LFQ min. ratio count was set to one. Peptides from both fractions were integrated in the LFQ intensity calculations. Only unique and razor peptides, unmodified or modified, were used for quantification. A list of all protein groups identified in CD14^+^ monocytes and THP-1 cells can be found in Supplementary Table S4. LFQ protein intensities were loaded into the Perseus framework (Max Planck Institute of Biochemistry)^41^. Known contaminants (human keratins, bovine and human plasma proteins) and reverse identified peptides/proteins were discarded. Intensities were log(2) transformed and missing values were imputed from the normal distribution of the data set (width: 0.3, downshift 2.4 for THP-1 cells and 1.8 for monocytes). Non-glycoproteins (uniprot annotation) were filtered out. Two- sample T-test (paired t-test for monocyte samples) was used to calculate statistical differences of protein abundances in the control and LPS treated groups. P-values were adjusted according to Benjamini and Hochberg and proteins demonstrating at least a two-fold expression difference and an adjusted p-value < 0.05 were considered to be significantly changed by LPS treatment. The mass spectrometry proteomics data have been deposited to the ProteomeXchange Consortium via the PRIDE^42^ partner repository with the dataset identifier PXD004034.

### Data Analysis

Protein groups identified by MaxQuant were filtered for proteins annotated as “glycoproteins” in the UniProt data base. Proteins annotated as transient O-GlcNac-modified without any other N- or O-glycosylation annotated were discarded. Data analysis was performed in R using packages provided by Bioconductor. The transmembrane hidden Markov model (TMHMM) algorithm (TMHMM 2.0 server)^43^ was used to predict putative TMDs in identified annotated glycoproteins. For gene ontology classification the R-package “org.Hs.eg.db” version 3.1.2 was used and results were manually compiled. Functional annotation clustering was performed with DAVID^44^, Version 6.7.

## Results

### Reduction in TNFα Production after 24 h and 48 h of LPS Pretreatment in Endotoxin Tolerant Cells

**Figure 1:**
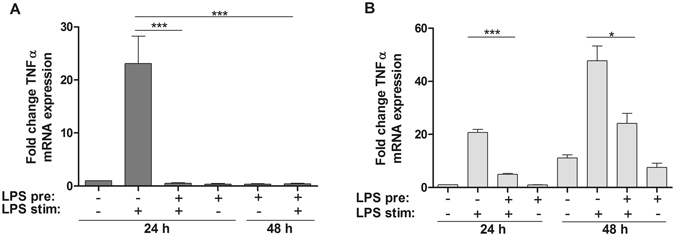
TNFα mRNA expression in naïve and LPS-reprogrammed monocytes indicates tolerance. Monocytes (**A**) and THP-1 cells (**B**) were pretreated for 24 or 48 h with fresh medium or 50 ng/ml LPS in fresh medium, washed, and either left untreated or re-challenged with 50 ng/ml LPS for 2 h. RNA was isolated, reversed transcribed, and analyzed by RT-qPCR. Shown are fold changes of TNFα mRNA production normalized to the house-keeping gene PPIB and compared to unstimulated control samples (mean of three independent experiments (THP-1) or three independent donors (monocytes) ± SE). ***p ≤ 0.001, *p ≤ 0.05.

To induce endotoxin tolerance, monocytes and THP-1 cells were treated with 50 ng/ml LPS for 24 h and 48 h and, subsequently, left untreated or were re-stimulated with 50 ng/ml LPS for 2 h. Control cells were either not stimulated (negative control) or challenged with LPS for 2 h (positive control) without pre-treatment. As shown by others^45–47^, assessment of TNF-α mRNA as a marker for the tolerant state revealed very low expression levels of this pro-inflammatory cytokine in the unstimulated control cells (Fig. 1A and B). LPS treatment of naïve cells for 2 h resulted in a strong upregulation of TNF-α mRNA in both cell types. Two hours of re-stimulation after 24 h and, likewise, after 48 h of pre-stimulation resulted in a pronounced attenuation of TNF-α mRNA production in monocytes and THP-1 cells, indicating that the cells entered the transient state of endotoxin tolerance.

### Glycoprotein Enrichment of LPS Stimulated THP-1 Cells

Next, we used hydrazide-based enrichment of glycoproteins from unstimulated or LPS stimulated cells to analyze differential glycoprotein expression in the tolerant state after 24 h and 48 h of LPS treatment. In addition, we included a time point zero (t = (0)) control and a 4 h treatment control in THP-1 cells. After treatment and cell lysis with SDS and boiling, glycoproteins were enriched and two peptide fractions derived from an on-bead tryptic digestion (tryptic peptides of glycoproteins, TPG fractions) and a second fraction consisting of PNGase F-released peptides (former N-glycopeptides, NGP fraction) were analyzed by LC-MS/MS for each biological sample. Peptide identifications and protein expression analysis was done with MaxQuant. Combined, the analysis of NGP and TPG fractions of all stimulations and time points resulted in the identification of 1179 and 1189 annotated glycoproteins detected in CD14^+^ monocytes and THP-1 cells data sets, respectively. TPG fractions contributed slightly higher numbers of protein group identifications compared to NPG fractions (Supplementary Fig. S1) but both fractions contributed high numbers of protein identifications and most glycoproteins were identified by peptides derived from both fractions.

### Glycoprotein Enrichment Identifies Soluble and Membrane-Anchored Proteins from Different Organelles

**Figure 2:**
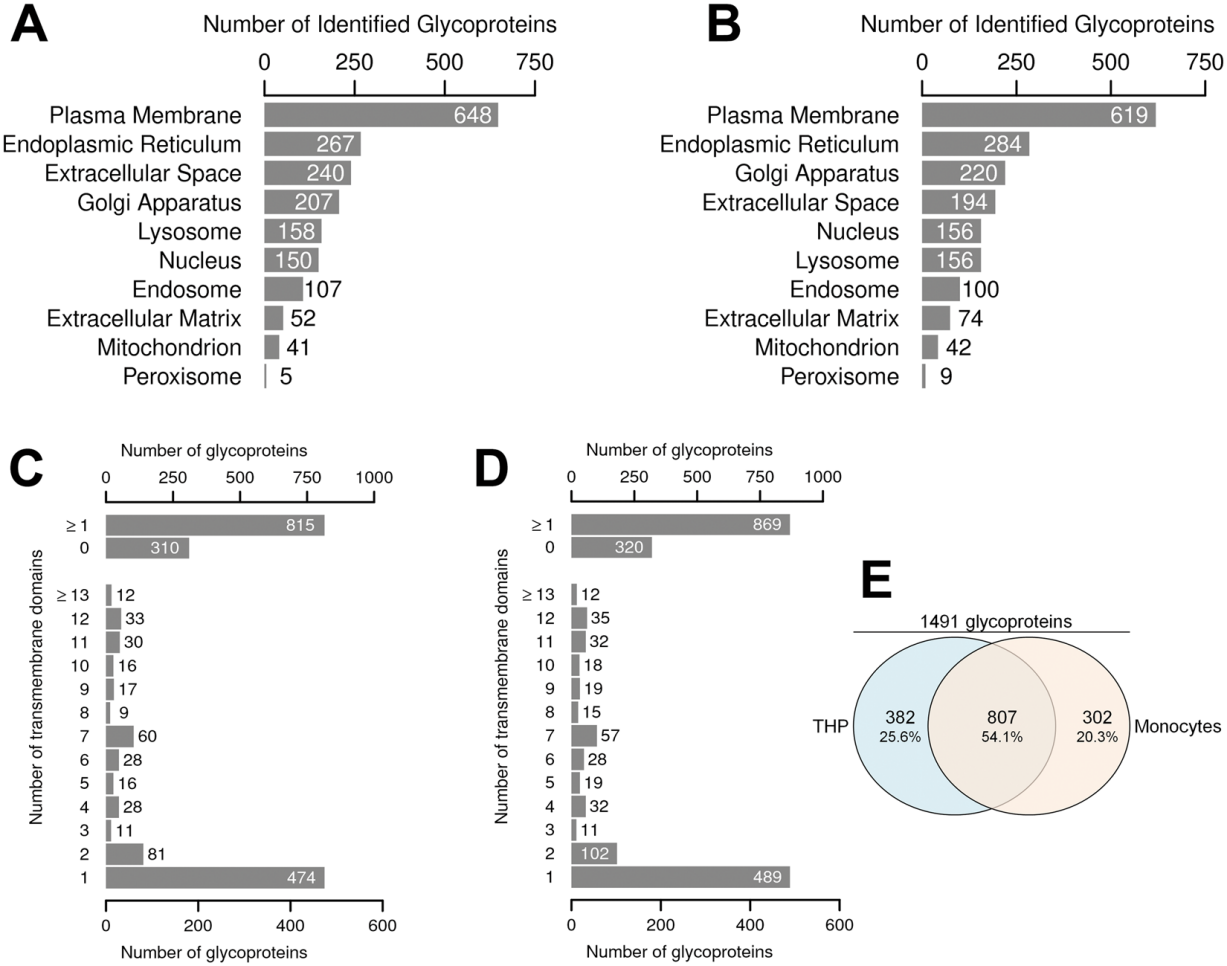
Glycoprotein identification in CD14^+^ monocytes and THP-1 cells by LC-MS/MS. (**A,B**) Gene ontology-based cellular component analysis of all glycoproteins identified in CD14^+^ monocytes (**A**, unique glycoproteins + soluble isoforms) and THP-1 cells (**B**, unique glycoproteins). Numbers of glycoproteins in manually selected cellular components are given. (**C,D**) Distribution of glycoproteins identified in CD14^+^ monocytes (**C**) and THP-1 cells (**D**) with predicted transmembrane helices identified in the two data sets. Upper part: glycoproteins with ≥1 or 0 predicted transmembrane helices, lower part: distribution of glycoproteins according to the number of predicted transmembrane domains. (**E**) Overlap between the 1491 annotated unique glycoproteins identified in THP-1 cells (1189) and CD14^+^ monocytes (1109).

Next, we examined from which cellular compartments the identified glycoproteins were derived. Subcellular compartment analysis revealed that in both cell types a major subset of 648 (monocytes) and 619 (THP-1 cells) identified glycoproteins was annotated in Gene Ontology (GO) as “plasma membrane” associated (Fig. 2A and B). Furthermore, taking into account the non-exclusive localization in GO and that only 52% of the N-glycoproteome is located outside the cell or beyond the plasma membrane^10^, we also identified glycosylated proteins derived from intracellular locations of cellular membrane coated organelles. The “endoplasmatic reticulum” and the “golgi apparatus” from both cell types contributed large and comparable numbers, ranging from 207–284 glycoprotein identifications. Slightly more glycoproteins annotated as “extracellular space” were identified in monocytes (240) compared to THP-1 cells (194), while glycoproteins annotated derived from “nucleus”, “lysosome”, “endosome”, “extracellular matrix”, “mitochondria”, and “peroxisome” were lesser in quantity but detected at similar numbers. To investigate whether the SDS-mediated cell lysis successfully solubilized transmembrane spanning proteins expressed by both cells, we further analyzed the list of identified glycoproteins for their transmembrane domain (TMD) numbers, using a hydrophobic transmembrane helix prediction algorithm (TMHMM v2.0 Server)^43^. The results show that in the data sets from CD14^+^ monocytes and THP-1 cells 815 and 869 glycoproteins, respectively, were predicted to contain at least one TMD (Fig. 2C and D). The majority of transmembrane domain containing proteins comprised a single predicted TMD, but glycoproteins comprising two or more predicted TMD were also identified to substantial numbers. Altogether, in both cell types 1491 unique annotated glycoproteins were identified, but only 807 glycoproteins (54.1%) were consistently detected in both data sets from unstimulated and LPS stimulated THP-1 cells and CD14^+^ monocytes (Fig. 2E), indicating large qualitative differences in glycoprotein expression between CD14^+^ monocytes and THP-1 cells.

### LPS Induced Glycoprotein Expression Changes in the Tolerant State

**Figure 3:**
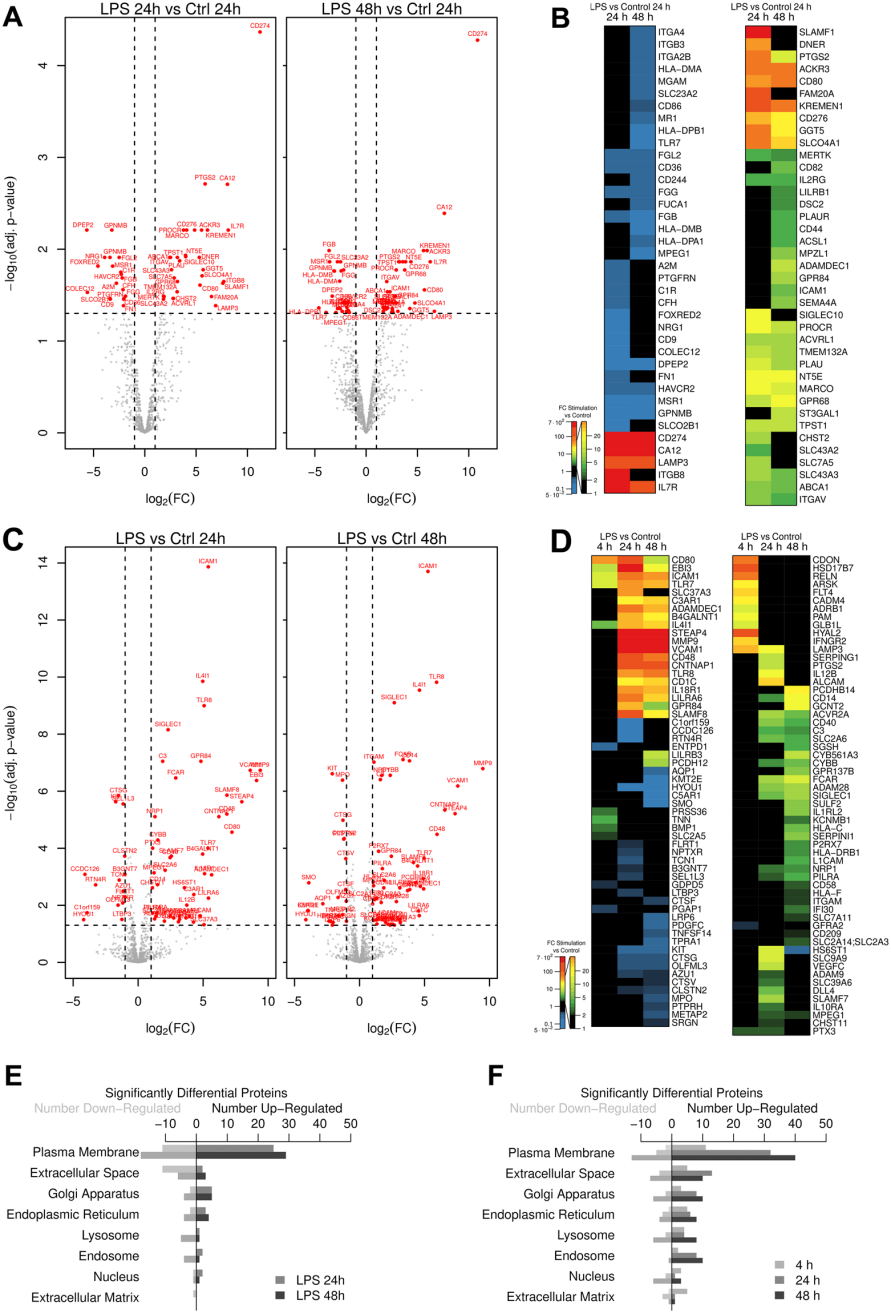
LPS regulated glycoproteins in CD14^+^ monocytes and THP-1 cells. (**A,B**) Volcano plot (**A**) and heat map (**B**) of differentially expressed glycoproteins in CD14^+^ monocytes. (**C,D**) Volcano plot (**C**) and heat map (**D**) of differentially expressed glycoproteins in THP-1 cells. (**A,C**) Volcano plots show the t-test p-value plotted against the glycoprotein expression fold change of all identified glycoproteins in CD14^+^ monocytes and THP-1 cells at 24 h (left) and 48 h (right) of LPS treatment. Data points in lower center area of the plots (grey) display unchanged or glycoproteins with no significant fold change, whereas data points in the upper left and upper right quadrants indicate glycoproteins (red) with significant negative (left) and positive (right) changes in protein abundances, respectively. For illustration gene names are given. (**B,D**) Black boxes in heat maps indicate no fold change at the given time point. Green, yellow, red indicate upregulation and blue indicates down regulation according to the legend. For illustration gene names are given. (**E,F**) Numbers of significantly changed glycoproteins after LPS treatment in selected cellular components at different time points in CD14^+^ monocytes (**E**) and THP-1 cells (**F**); 4 h = light grey bars, 24 h = dark grey bars, and 48 h = black bars.

For quantitative analysis, the proteomes of enriched glycoproteins of untreated cells were compared to LPS treated samples at the different time points investigated. In the monocyte data set, both, the 24 h and 48 h LPS treated samples were compared to the unstimulated 24 h control samples, due to marked decreased cell numbers and protein identifications in the 48 h cultures of unstimulated monocytes, making these samples unsuitable for normalization and statistical analysis. CD14^+^ monocytes activated by LPS in the tolerant state displayed 19 significantly downregulated and 32 significantly upregulated glycoproteins at 24 h of LPS treatment, and 24 downregulated and 36 upregulated glycoproteins at 48 h (Fig. 3A, volcano plots and Fig. 3B, heat map). In total, a significant change in protein abundance was detected for 77 glycoproteins in the monocyte data set, 34 glycoproteins displayed reduced and 43 glycoproteins increased expression levels after LPS treatment. No significant protein expression changes were detected when we compared 24 h and 48 h stimulated CD14^+^ monocytes (Supplementary Fig. S1), demonstrating that the majority of protein expression changes occurred already early in the LPS time course, though, not all fold changes reached statistical significance at 24 h or 48 h. At 24 h and 48 h of LPS treatment, 9 and 34 glycoproteins were consistently down- or upregulated, respectively. Fold changes and p-values of all identified and significantly regulated glycoproteins in CD14^+^ monocytes can be found in Supplementary Table S3, and a comparison of fold changes of significant regulated glycoproteins in CD14^+^ monocytes compared to the expression changes identified in THP-1 cells can be found in Supplementary Table S2. Among the strongest upregulated glycoproteins at 24 h and 48 h were two well-known NFκB-dependent LPS upregulated molecules, costimulatory B7-family members programmed cell death-1 ligand-1 (PDL1/CD274) and CD276-antigen^48, 49^, while the expression level of the membrane-anchored components of the LPS receptor complex, CD14 and TLR4, were not affected.

In THP-1 cells, short-term LPS treatment for 4 h resulted in the identification of only 26 glycoproteins significantly up- or downregulated at the protein level (Supplementary Fig. S1, and Fig. 3D, heat map - left panel). Intercellular adhesion molecule 1 (ICAM-1), a Nuclear-factor-κ-B (NFκB)-regulated cell surface expressed glycoprotein early upregulated in response to LPS treatment of THP-1 cells^50^ was evident among the early upregulated proteins. During the LPS time course of THP-1 cells, glycoprotein numbers regulated by LPS increased steadily. At 24 h and 48 h of stimulation 62 and 74 glycoproteins were differentially expressed (Fig. 3C and D), respectively. The majority of LPS regulated glycoproteins at 24 h and 48 h displayed increased expression levels compared to unstimulated control samples, and only 15 and 21 glycoproteins at 24 h and 48 h, respectively, demonstrated a significant downregulation at these later time points. Most of the downregulated glycoproteins at 24 h of LPS treatment showed a transient expression change, matching again the expression level in the control samples at 48 h.

### Glycoprotein Expression Changes in Cellular Organelles

In a next step, we analyzed whether LPS treatment of CD14^+^ monocytes and THP-1 cells affected glycoprotein expression in all cellular organelles or if specific intracellular organelles were particularly affected. Most of the differentially expressed glycoproteins were annotated as associated with the plasma membrane, accounting for 8–9% of all plasma membrane annotated proteins in both, CD14^+^ monocytes and THP-1- cells (Fig. 3E and F). Similar percentages of regulated glycoproteins were also found for the term “Extracellular space” (7–8%). Interestingly, compared to CD14^+^ monocytes THP-1 cells displayed a greater number of upregulated glycoproteins in the categories “extracellular space”, “lysosome” and “endosome”. Glycoproteins localizing to “Golgi apparatus” (6.8% in THP-1 and 2.4% in monocytes), “Endoplasmic reticulum” (4% in THP-1 and 2% in monocytes) were also partially affected. The data demonstrate that an altered glycoprotein expression was found in all intracellular organelles but the “plasma membrane” and the “extracellular space” categories were most strongly affected, especially at late time points during the LPS time course.

### Comprehensive Analysis of CD Antigen Expression Changes in Response to LPS

**Figure 4:**
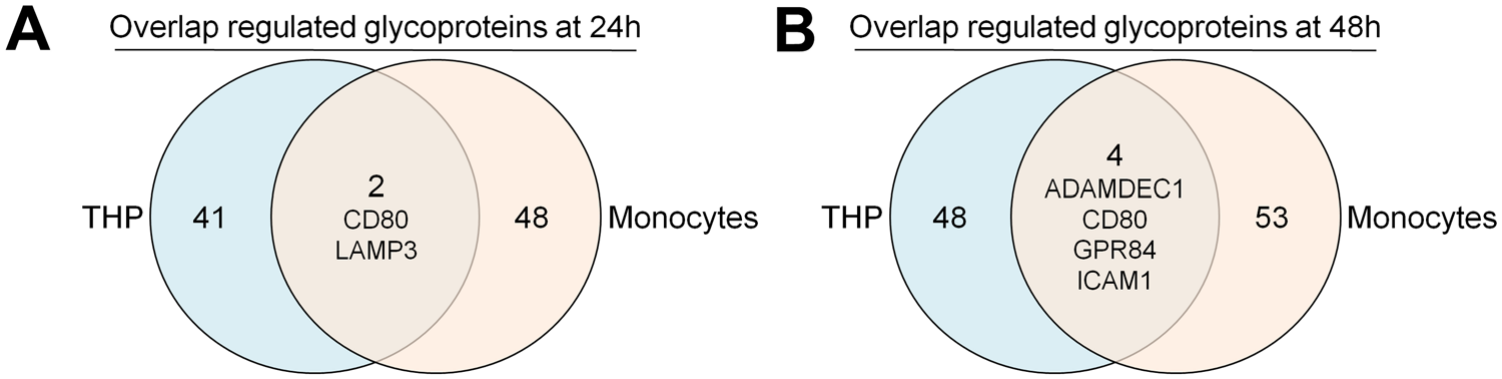
Comparison of differently expressed glycoproteins identified in CD14^+^ monocytes and THP-1 cells. (A + B) Venn diagrams showing the overlap between differently expressed glycoproteins identified in monocytes and THP-1 cell at 24 h (**A**) and 48 h (**B**) of LPS stimulation.

Primarily affected by LPS treatment were expression levels of plasma membrane associated glycoproteins. Thus, we performed a multiplexed proteomic cell surface-associated CD antigen phenotyping to distinguish the CD antigen expression profile of unstimulated and LPS stimulated cells in the tolerant state. In total, we identified 189 CD antigens in the CD14^+^ monocyte data set (Supplementary Fig. S2) and 180 CD antigens in the complete data set of THP-1 cells (Supplementary Fig. S3). T-cell (CD3) and B-cell markers (CD19) were not identified in the monocyte data set. Neutrophil markers (CD66b and CCD66a) were identified, indicating small impurities with this cell type, but did not change in abundance at the time points investigated. Differential expression was detectable for 26 and 24 CD antigens in CD14^+^ monocytes and THP-1 cells, respectively. In both cell types, the majority of LPS regulated CD antigens displayed an upregulated protein expression. Interestingly, of all identified CD antigens only upregulated CD54/ICAM-1, CD80, and LAMP3/CD208 demonstrated a consistent expression change in both cell types. All other differentially expressed glycoproteins in CD14^+^ monocytes were either not identified or demonstrated an unchanged, or not significantly altered, expression level in THP-1 cells. Therefore, we analyzed the identified expression changes on the global level to reveal similar glycoprotein expression changes in LPS-tolerized CD14^+^ monocytes and THP-1 cells. Though, comparable numbers of differentially expressed glycoproteins in CD14^+^ monocytes and THP-1 cells at 24 h (43 and 50 in THP-1 and monocytes, respectively) and 48 h (53 and 58 in THP-1 cells and monocytes, respectively) were identified, the overlap at both time points was only marginal. After 24 h of LPS stimulation (Fig. 4A) only CD54 and CD80 were consistently found differentially expressed in both cell types and after 48 h (Fig. 4B) four differentially expressed glycoproteins were identified in both cell types (ADAMDEC1, CD80, GPR84, and ICAM1).

### LPS Induced Expression Changes of Enzymes Involved in Protein Glycosylation

To investigated whether LPS treatment altered the expression level of glycosylated enzymes involved in glycan maturation that might have affected our protein enrichment via carbohydrate structures and the quantitation on protein level, protein abundance changes in the CD14^+^ monocyte and THP-1 cell and data sets for the GO-category GO:0006486 “protein glycosylation” was analyzed. We identified 76 and 92 glycoproteins in CD14^+^ monocyte and THP-1 cells, respectively (Supplementary Figures S4 and S5). Of the identified glycoprotein enzymes involved at the various steps of protein glycosylation one protein (ST3GAL1) was upregulated in CD14^+^ monocytes and four proteins (B3GNT7, B4GALNT1, CCDC126 and GCNT2) exhibited detectable abundance changes in THP-1 cells. Only GCNT2, upregulated in THP-1 cells after 48 h exhibit broad substrate specificity and could have affected our glycoproteomics approach by increasing the branching degree of glycoconjugates attached to glycoproteins. Therefore, we tested whether the branching degree of THP-1 cell surface exposed glycans was significantly altered after LPS treatment by binding of FITC-conjugated Phytohemagglutinin-L (PHA-L) lectin from *Phaseolus vulgaris* with high affinity towards 2,6-branched tri- and tetra-antennary complex-type N-glycans^51^. Analysis by flow cytometry revealed high PHA-L binding at all time points and no detectable changes in cell surface associated branched glycan structures (Supplementary Fig. S6).

### Expression of G protein-Coupled Receptors after LPS Treatment

As we were interested in the identification of new possible drug targets expressed on the cell surface of tolerant monocytes to interfere with the tolerant state, we next analyzed the expression level of G-protein-coupled receptors (GPCRs), a large family of N-glycosylated seven-transmembrane domain receptors, in more detail. In the CD14^+^ monocyte data set 52 proteins with G-protein coupled receptor activity were identified and three receptors, ACKR3, GPR68 and GPR84 revealed statistical significant higher expression levels during the LPS time course (Supplementary Fig. S6). In the THP-1 glycoproteome data set we identified 53 proteins annotated with G-protein coupled receptor activity (GO:0004930) (Supplementary Fig. S7) and six GPCRs demonstrated an altered expression in response to LPS. Only GPR84 demonstrated upregulated expression in both, CD14^+^ monocytes and THP-1 cells, significant differentially expressed at 48 h of LPS stimulation.

### Verification of Glycoprotein Expression Changes

**Figure 5:**
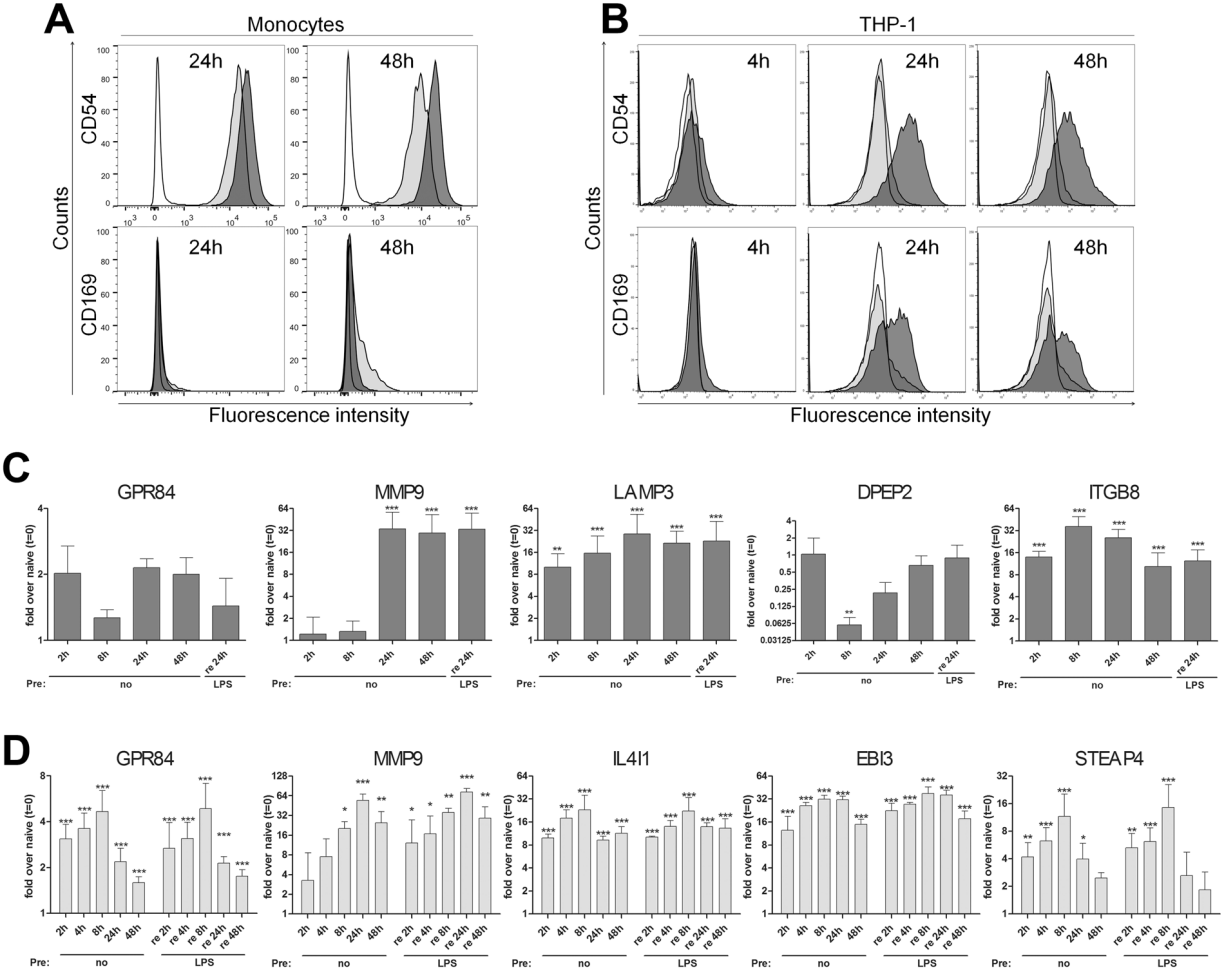
Verification of proteomic results by flow cytometry and qPCR. Cell surface expression of ICAM1/CD54 and SIGLEC1/CD169 on CD14^+^ monocytes (**A**) and THP-1 cells (**B**). (**A**) Monocytes were either left untreated or stimulated for 24 h or 48 h with 50 ng/ml LPS. White graphs: isotype controls, in light grey: expression at the indicated time point of unstimulated controls, in dark grey: expression at the indicated time points after LPS treatment. The data shown are representative of two different donors independently analyzed. Depicted are the intensity levels of the indicated proteins expressed on the CD14^+^ cell population (**B**) THP-1 cells were either left untreated or stimulated with 50 ng/ml LPS for 4 h, 24 h, and 48 h. White graphs: expression in unstimulated controls at t = (0), in light grey expression at the indicated time point of unstimulated controls, in dark grey: expression at the indicated time points after LPS treatment. The data shown are representative of three independent experiments. (**C,D**) Gene expression changes of selected glycoproteins induced by LPS treatment revealed by qPCR. (**C**) Monocyte mRNA expression changes of GPR84, MMP9, LAMP3, DPEP2, and ITGB8 at different time points after LPS treatment of naïve (pre: no), or 24 h LPS pre-stimulated (pre: LPS) monocytes re-stimulated with LPS for the indicated time points. (**D**) THP-1 mRNA expression changes of GPR84, MMP9, IL4i1, EBI3, and STEAP4 at different time points after LPS treatment of naïve (pre: no), or 24 h LPS pre-stimulated (pre: LPS) THP-1 cells re-stimulated with LPS for the indicated time points. (C + D) Plotted are fold changes normalized to the house-keeping gene PPIB (monocytes) or HPRT (THP-1) and compared to naïve unstimulated control cells at t = (0). Samples (mean of three independent experiments (THP-1 cells) or three independent donors (monocytes) ± SE) *p < 0.05, **p < 0.01, ***p < 0.001.

Upregulation of cell surface associated ICAM1 on CD14^+^ monocytes and on THP-1 cells was confirmed by flow cytometry (Fig. 5A and B, upper panel). Additionally, the increased abundance of SIGLEC1 after LPS treatment was confirmed for THP-1 cells (Fig. 5B, lower panel). In CD14^+^ monocytes SIGLEC-1 demonstrated a downregulation after 24 h and 48 h of LPS treatment in the glycoproteomic analysis, not reaching statistical significance at either time point. Nevertheless, a small subpopulation of naïve CD14^+^ monocytes expressed SIGLEC-1 on the cell surface, and expression was reduced after LPS treatment at 24 h and, more pronounced, also at 48 h (Fig. 5A, lower panels). In addition, upregulated cell surface expression after 24 h and 48 h of LPS treatment was confirmed by flow cytometry for CD80, CD274, CD80, CD127/IL7R, CD319/SLAMF7 (Supplementary Fig. S8). Downregulation of CD86 and also of CLEC12A, a C-type-lectin family member with decreased expression level not reaching statistical significance in the proteomic analysis were also confirmed (Supplementary Fig. S8). Monocytes analyzed by qPCR (Fig. 5C) demonstrated significantly upregulated mRNA expression of the glycoproteins LAMP3 and ITGB8, already detectable at 2 h of LPS treatment. MMP9 mRNA was significantly upregulated at later time points, at 24 h and 48 h of stimulation, confirming the proteomic results. DPEP2 was found significantly reduced on protein level in CD14^+^ monocytes after LPS treatment and DPEP2 mRNA expression analysis by qPCR demonstrated a transient reduced expression of this gene, significant after 8 h. mRNA levels of GPR84, significantly upregulated on protein level after 48 h of LPS treatment demonstrated slightly higher mRNA expression levels in LPS treated cells during the whole time course, though significance was not reached at the time points investigated. Restimulation of 24 h pre-stimulated cells with an additional LPS challenge for 24 h did not change the mRNA level of all genes analyzed in comparison to the 48 h LPS stimulated cells. Additionally, to confirm the glycoproteomics results of THP-1 cells, we also examined the demonstrated upregulation of MMP9, STEAP4, EBI3, IL4i1 and GPR84 in the glycoproteomic analysis on mRNA level by qPCR (Fig. 5D). LPS treatment of THP-1 led to increased mRNA production of MMP9, STEAP4, EBI3, IL4I1 and GPR84 detectable already two hours after stimulation. Next, we also tested whether the activation of gene transcription of MMP9, STEAP4, EBI3, IL4I1 and GPR84 was affected in the tolerant state. Analysis of the kinetics of mRNA production after LPS re-stimulation revealed that in contrast to TNFα gene expression, mRNA production encoding MMP9, STEAP4, EBI3, IL4i1 and GP84 was not affected by LPS pre-stimulation and all five genes were re-expressed with the same kinetics and expression level in the tolerant state.

### Treatment of LPS Pre-stimulated Cells with Decanoic Acid Increases TNFα mRNA Expression

**Figure 6:**
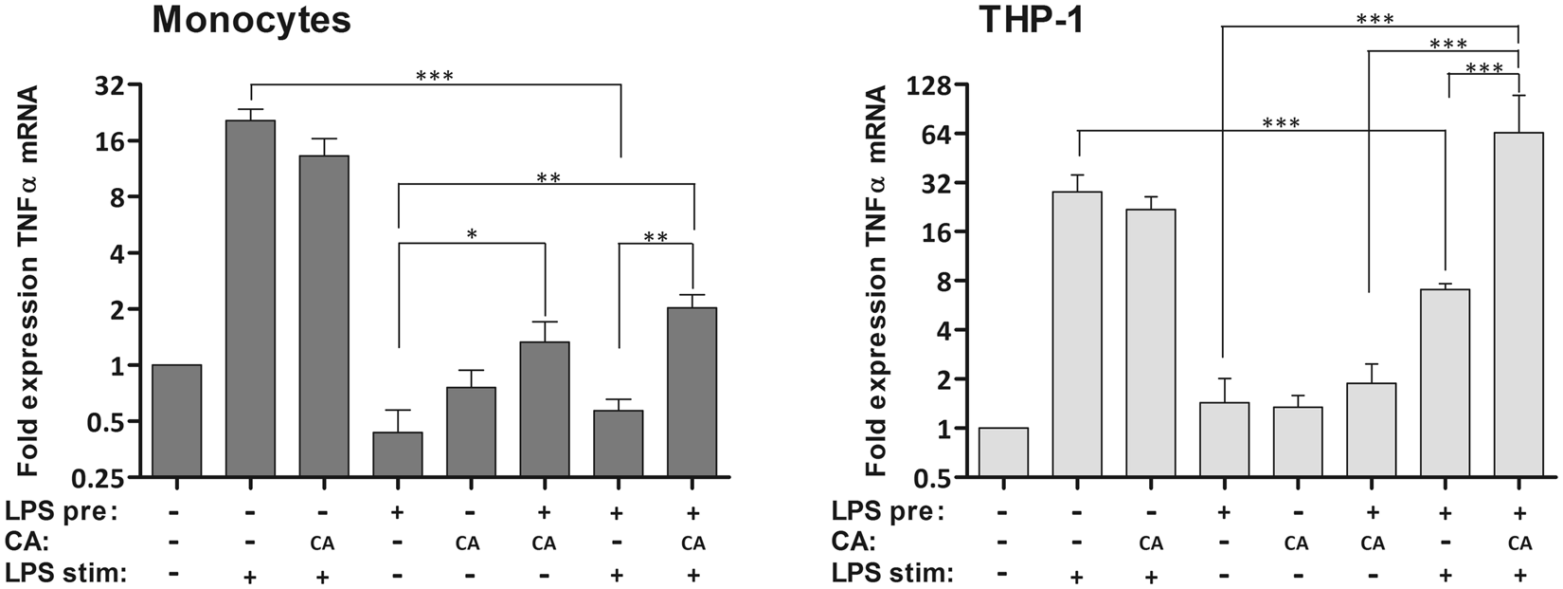
TNF-alpha mRNA production in the LPS pre-stimulated tolerant state is modulated by Capric acid treatment. (**A,B**) Monocytes (**A**) and THP-1 cells (**B**) were pretreated for 24 h with fresh medium or 50 ng/ml LPS in fresh medium. Two hours after the initial stimulation Capric acid (500 µM) was added to some samples (CA). After 24 h cells were re-stimulated with 50 ng/ml LPS for 2 h or left untreated. RNA was isolated, reversed transcribed, and analyzed by RT-qPCR with primers specific for PPIB and TNFα. Shown are the fold changes of TNFα mRNA normalized to the house-keeping gene PPIB (monocytes) or HPRT (THP-1 cells) and compared to unstimulated control samples (mean of three independent experiments (THP-1 cells) or 5 different donors (monocytes) ± SE). *p ≤ 0.05; **p ≤ 0.01; ***p ≤ 0.001.

GPR84 is a receptor for free fatty acids and can be potently activated by saturated medium-chain free fatty acids (MCFAs) like Decanoic acid (Capric acid, C10:0), undecanoic acid (C11:0) and lauric acid (C12:0)^52^. Increased receptor expression in activated macrophages and monocytes was found^52^ and confirmed on protein level in this study. Recent reports have demonstrated that GPR84 activation with MCFAs can amplify inflammatory responses elicited by LPS, like chemotaxis and production of pro-inflammatory cytokines^53^. Therefore, we wondered if GPR84 activation with Capric acid would be able to influence pro-inflammatory TNFα mRNA expression in the tolerant cell state after re-stimulation with LPS. Addition of Capric acid to unstimulated monocytes and THP-1 cells for 20 hours before LPS treatment did not influence TNFα mRNA production in the pro-inflammatory response to LPS (Fig. 6A and B). Furthermore, Capric acid treatment alone, without LPS stimulation, did not alter TNFα mRNA production in both cell types. Addition of Capric acid 2 h after the initial LPS pre-stimulation increased slightly the TNFα mRNA long term (24 h) expression in monocytes, while THP-1 cells were not affected. In contrast, addition of Capric acid to LPS pre-stimulated cells led to slight, but significant increased production of TNFα mRNA in monocytes (Fig. 6A) after re-stimulation with LPS in the tolerant state and to strongly increased production of TNFα mRNA in THP-1 cells (Fig. 5A), comparable to the pro-inflammatory TNFα production of naive THP-1 cells.

## Discussion

Endotoxin tolerance is a mechanism for attenuation of exuberant immune responses and is defined as transient unresponsiveness to repeated doses of LPS or other PAMPs, accompanied with specific alterations in gene transcription mediated by epigenic modifications^54^ and altered signaling pathways downstream of TLR activation^27^. Although endotoxin tolerance can be beneficial for the infected host serving as an important mechanism for protection against endotoxin shock and extensive tissue damage, the tolerant cell state of monocytes and macrophages after severe systemic infections like sepsis is also linked to high risk of secondary infections and mortality^24^. Only few studies demonstrated a direct involvement of membrane spanning proteins and other glycoproteins in regulatory mechanisms leading to impaired TLR signaling or endotoxin development^55–57^, and a cell surface protein signature to distinguish tolerant monocytes from naïve cells is wanting. In the current study, we comprehensively analyzed glycoprotein expression changes in LPS stimulated peripheral blood CD14^+^ monocytes and monocytic THP-1 cells to distinguish naïve cells from activated cells in the tolerant state and to identify glycoproteins that can be targeted by drug treatment to modulate the tolerant cell state. Hydrazide-chemistry-assisted solid phase extraction of whole glycoproteins^9^ resulted in the identification of more than 1100 glycoproteins in the data sets of both cell types and the majority of identified glycoproteins were transmembrane domain containing proteins from various cellular organelles. Quantitative analysis of the CD14^+^ monocyte glycoproteome and of the THP-1 glycoproteome at different time points after LPS challenge revealed significantly altered expression levels of 7 and 10% of all identified glycoproteins in CD14^+^ monocytes and THP-1 cells, respectively. Differentially expressed glycoproteins originated from all intracellular membrane coated compartments and comprised membrane-anchored, secreted or soluble glycoproteins. Qualitatively, the global glycoproteomes of CD14^+^ monocytes and THP-1 cells resembled each other to only 54.1%, and differentially expressed glycoproteins in the tolerant state differed considerably.

Several findings from other groups are in concordance with the tolerant monocyte glycoprotein signature identified by our proteomic approach. Allam *et al*. demonstrated an upregulation of CD274 and CD276 and a downregulation of CD86 by TLR4-activation of human oral Langerhans cells that released predominantly anti-inflammatory cytokines and showed decreased stimulatory capacity towards T-cells after activation^58^. CD86 downregulation on monocytes was also demonstrated by Wolk *et al*.^31^ and in the same study the authors demonstrated the upregulated expression of CD54 and CD80 in endotoxin tolerant monocytes with impaired antigen presentation, which were also upregulated in our proteomic analysis. Furthermore, we observed significant reduced expression of several HLA-molecules (HLA-DMA, HLA-DMB, HLA-DPA1, and HLA-DPB1) in the CD14^+^ monocyte data set, in particular after 48 h of LPS stimulation. Downregulation of HLA class II genes in tolerant monocytes was reported in *in vitro* models, in reprogrammed monocytes from cystic fibrosis patients, in patients with septic shock, and in tumor-tolerized monocytes^20, 28, 31, 59^ and is considered as a hallmark of the reduced antigen presentation capacity of monocytes in the tolerant cell state. Decreased expression of HLA-DR molecules, the current gold standard for the detection of monocyte tolerance by cell surface expressed proteins on monocytes^60^, was found in the CD14^+^ monocyte data set, though, without statistical significance. This indicates that the *in vitro* analysis of purified monocytes in culture as a model for monocyte phenotyping in the tolerant state might not reflect all proteomic changes that can occur *in vivo*, when the diversity of soluble mediators released by other myeloid cells or tissues react on peripheral blood monocytes. Nevertheless, we also detected the previously reported upregulation of non-tolerizable genes^20, 54^ on the glycoprotein level, e.g. Prostglandin G/H synthase 2 (PTGS2) and scavenger receptor MARCO in our CD14^+^ monocyte data set, further confirming previously published observations. The anti-microbial receptors TREM1, CLEC4A and FPR1 were slightly upregulated but the fold changes were either below the 2-fold threshold, or did not reach statistical significance. Plasma membrane associated negative regulatory molecules of TLR signaling, SIGIRR and CEACAM1, were consistently detected and unchanged in expression after LPS treatment. In addition, the only immunoreceptor tyrosine-based inhibitory motif-comprising receptor upregulated in the tolerant state was LILRB1, statistical significant after 48 h, indicating that the reprogramming of monocytes after LPS was not associated with a massive upregulation of plasma membrane associated negative signaling regulators contributing to monocyte tolerance.

Upregulated and downregulated glycoproteins in CD14^+^ monocytes after 24 h and 48 h of LPS treatment identified in this study represent likely candidates to characterize the tolerant reprogrammed cell state and might be tested in future studies with cells from patients with ET-related pathologies. Among the significantly regulated proteins in CD14^+^ monocytes identified after 24 h and 48 h of LPS treatment, LAMP3, IL7R and ITGB8 were detected with highly increased expression in the tolerant state, represent interesting marker candidates with additional putative functions in reprogrammed monocytes. LAMP3, or DC-LAMP, also upregulated in the tolerant state of THP-1 cells, localizes predominantly to lysosomes where it co-localizes with MHC II compartments in human dendritic cells^61^. LAMP3 might be involved in the transfer of peptide-MHC II molecule complexes to the cell surface^61, 62^, and, therefore, might be a marker for the reduced expression of HLA molecules and decreased stimulatory capacity towards T-cells. IL7R expression in LPS activated monocytes was demonstrated on mRNA level by Larabee *et al*.^63^, and monocyte IL7R expression can be modulated by increased cAMP and also by Notch signaling mechanisms. IL7R is the receptor for interleukin-7 (IL-7) or, together with cytokine receptor-like factor 2 (CRLF2 or TSLPR), forms the heterodimeric receptor complex for thymic stromal lymphopoeitin (TSLP). CRLF2 expression was slightly increased in LPS-tolerized CD14^+^ but the expression change was not statistically significant in the glycoproteomic study. Nevertheless, LPS-activated monocytes express receptors for IL-7 and TSLP, two signaling molecules involved the regulation of myeloid cells during sepsis pathology^64, 65^. TSLP level in plasma are early upregulated in septic mice and TSLP signaling via the TSLPR was associated with improved survival and a reduction of the pro-inflammatory cytokine response^64^. IL-7, on the other hand, was shown to ameliorate immune dysfunction in sepsis^65^ and is discussed and currently studied for immunoadjuvant therapy^24^. Most studies conducted to date concentrated on IL-7-mediated effects on restoring lymphocyte functionality during sepsis^65, 66^. With the presented results in this study, demonstrating that LPS-activated tolerant CD14^+^ monocytes upregulate IL7R on the cell surface, it can be speculated that IL-7 administration during sepsis will also affect the IL7R-positive tolerant monocyte population. ITGB8 was undetectable in naïve monocytes on protein level, and mRNA transcript level demonstrated a strong upregulation during the LPS time course. ITGB8 seems to be a good candidate for the identification and segregation of tolerant monocytes from naive monocytes. Furthermore, together with the alpha v-Integrin subunit, this heterodimeric integrin receptor is implicated in the extracellular activation of TGF-β from its latent complex, the central mediator of fibrosis in multiple organs. Inhibition and blockage of αVβ8-integrin results in a phenotype similar to all developmental effects of TGF-β1 and TGF-β3^67^ loss, and recently it was shown that blockage of αV-integrins attenuates fibrosis in several tissues^68^. Therefore, LPS-activated αVβ8-integrin-positve CD14^+^ monocytes might be involved in the activation of anti-inflammatory and pro-fibrotic TGF-β from latent TGF-β complexes in the blood stream and tissues, respectively.

GPR84, a receptor for medium-length free fatty acids^53^ was upregulated on protein level in LPS activated tolerant CD14^+^ monocytes and THP-1 cells. While receptor expression was undetectable in untreated THP-1 cells, monocytes expressed GPR84 already in the naïve state. GPR84 expression is mainly observed in bone marrow, lung, and peripheral blood leucocytes and was shown to increase in inflammatory conditions mediated by LPS or TNFα^52, 69^. MCFAs with a carbon chain length of 9–14 are native ligands and activate the receptor, transducing the signal to a pertussis toxin sensitive G_i/o_ pathway^52^, thereby amplifying LPS-mediated pro-inflammatory cytokine expression of e.g. IL12 p40, IL-8 and TNFα. Therefore, medium-chain FFAs might link fatty acid metabolism to immunological regulation and inflammatory diseases^69, 70^. Capric acid stimulation after the initial LPS treatment led to slightly increased TNFα mRNA expression in response to a subsequent LPS stimulus in monocytes and to restored TNFα mRNA expression in tolerant THP-1 cells, comparable to naïve cells. The data demonstrate that GPR84 and Capric acid can modulate the refractory cell state of monocytes by a mechanism that leads to increased transcription of a T-gene like TNFα. As GPR84 activation with MCFAs was demonstrated to amplify the LPS response of several other pro-inflammatory cytokines^53^ MCFAs and GPR84 might have the potential to modulate the tolerant state of monocytes more generally. For example in inflamed adipose tissue, GPR84 expression is increased^69^ and might contribute to sustained inflammation. Future studies addressing MCFA and GPR84 signaling in activated monocytes will offer valuable clues about the therapeutic potential and mechanistic insights to modulate the tolerant cell state of monocytes.

## Electronic supplementary material

Supplementary_Information_Mueller_MM

Supplementary Table S3

Supplementary Table S4
