# Supplementary material for: Global analysis of glycoproteins identifies markers of endotoxin tolerant monocytes and GPR84 as a modulator of TNFα expression
Source: Sci Rep. 2017 Apr 12;7:838. doi: 10.1038/s41598-017-00828-y (PMC5429802; doi:10.1038/s41598-017-00828-y)
Supplement: Supplementary file 1 — Supplementary_Information_Mueller_MM [file 41598_2017_828_MOESM1_ESM.pdf]

Global analysis of glycoproteins identifies markers of endotoxin tolerant monocytes and GPR84 as a modulator of TNFα expression

Mario M. Müller[1,4], Roland Lehmann[1], Tilman E. Klassert[1],Theresia Conrad[1,3],Stella Reifenstein[1], Christoph Moore[1], Anna Kuhn[1,3], Andrea Behnert[4], Reinhard Guthke[3], Dominik Driesch[2], and Hortense Slevogt[1,*]

[1]Septomics Research Center, Jena University Hospital, Jena, Germany
[2]BioControl Jena GmbH, Jena, Germany
[3]Leibnitz Institute for Natural Product Research and Infection Biology – Hans-Knöll-Institut, Jena, Germany
[4]Jena University Hospital, Integrated Research and Treatment Center - Center for Sepsis Control and Care (CSCC), Jena, Germany

SUPPLEMENTARY INFORMATION

Table of Contents:

Figure S1: Glycoprotein identifications in THP-1 cells and CD14$^+$ monocytes by LC-MS/MS.
Figure S2: Overview of the 189 identified glycoproteins representing CD antigens identified in the LPS time course in CD14$^+$ monocytes at time points 24h and 48h.

Figure S3: Overview of the 180 identified glycoproteins representing CD antigens identified in the LPS time course in THP-1 cells at time points 4h, 24h and 48h.

Figure S4: Overview of 92 glycoproteins annotated as being involved in "protein glycosylation" detected the time course in CD14$^+$ monocytes.

Figure S5: Overview of 92 glycoproteins annotated as being involved in "protein glycosylation" in the THP-1 data set and branching degree of cell surface glycan chains assessed by PHA-L staining and flow cytometry.

Figure S6: Overview of "G-protein-coupled receptor activity" annotated glycoproteins detected during the LPS time course in CD14$^+$ monocytes.

Figure S7: Overview of "G-protein-coupled receptor activity" annotated glycoproteins detected during the LPS time course in THP-1 cells.

Figure S8. Verification of proteomic results by flow cytometry (CD14$^+$ monocytes).

Table S1: Primer list.

Table S2: Comparison of FC determined for differentially expressed glycoproteins identified in CD14+ monocytes with the results from THP-1 cells.

Table S3: Table of all identified glycoproteins in unstimulated and LPS stimulated THP-1 cells and CD14$^+$ monocytes with fold changes and p-values.

Table S4: All Protein groups identified in THP-1 cells or CD14$^+$ monocytes with LFQ-values identified by MaxQuant.

Supplementary Figure 1

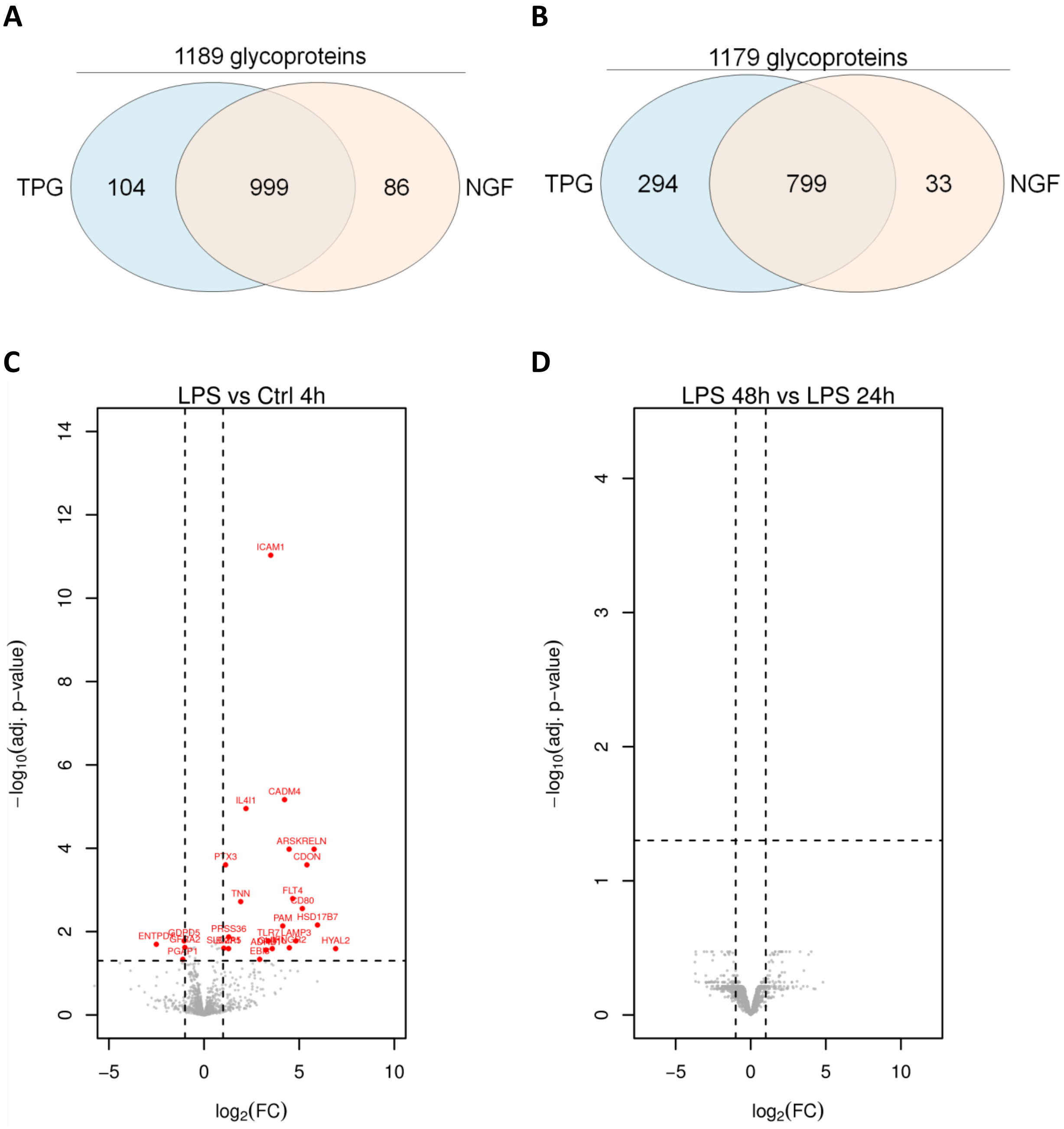

Supplementary Figure S1. Glycoprotein identifications in THP-1 cells and CD14^{+} monocytes by LC-MS/MS. (A) Overlap between glycoprotein identifications in the N-glycopeptide fraction (NGF, 1085 glycoproteins) and the tryptic peptide fraction of enriched glycoproteins (TPG, 1103) detected in the whole data-set of THP1 cells (1189 glycoproteins), or (B) monocytes (NGF 832 glycoproteins, TPG 1146 glycoproteins and whole data set 1179 glycoproteins). (C,D) Volcano plots showing the t-test p-value plotted against the glycoprotein expression fold change of (C) all identified glycoproteins at 4h in THP-1 cells and (D) comparison of all identified glycoproteins after 24h and 48h of LPS treatment in CD14^{+} monocytes (no significant change detectable).

Supplementary Figure S2

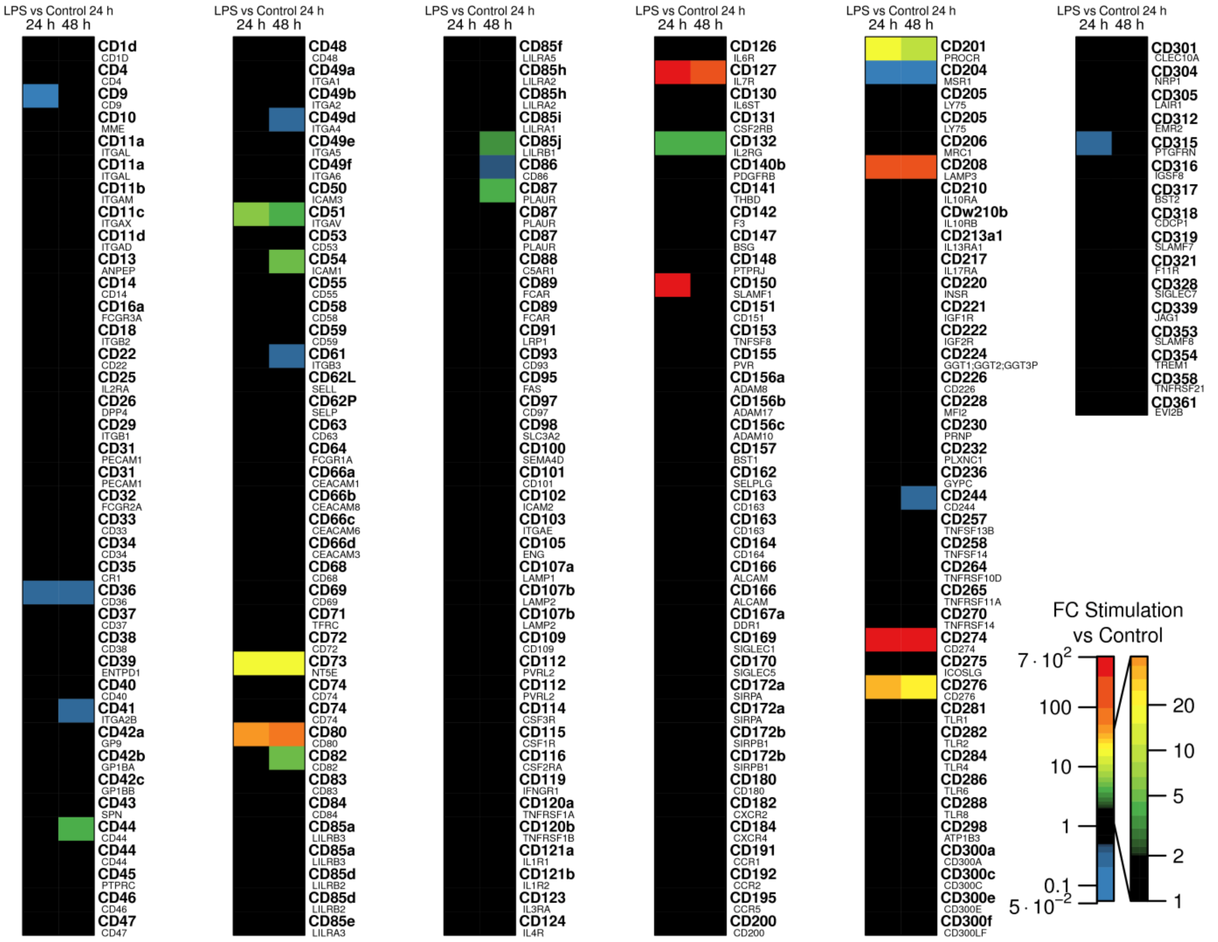

Supplementary Figure S2. CD antigens identified and regulated in CD14⁺ monocytes. Overview of the 189 identified glycoproteins representing CD antigens identified in the LPS time course in monocytes at time points 24h and 48h. The color denotes significant up- or downregulation by LPS treatment according to the legend.

Supplementary Figure S3

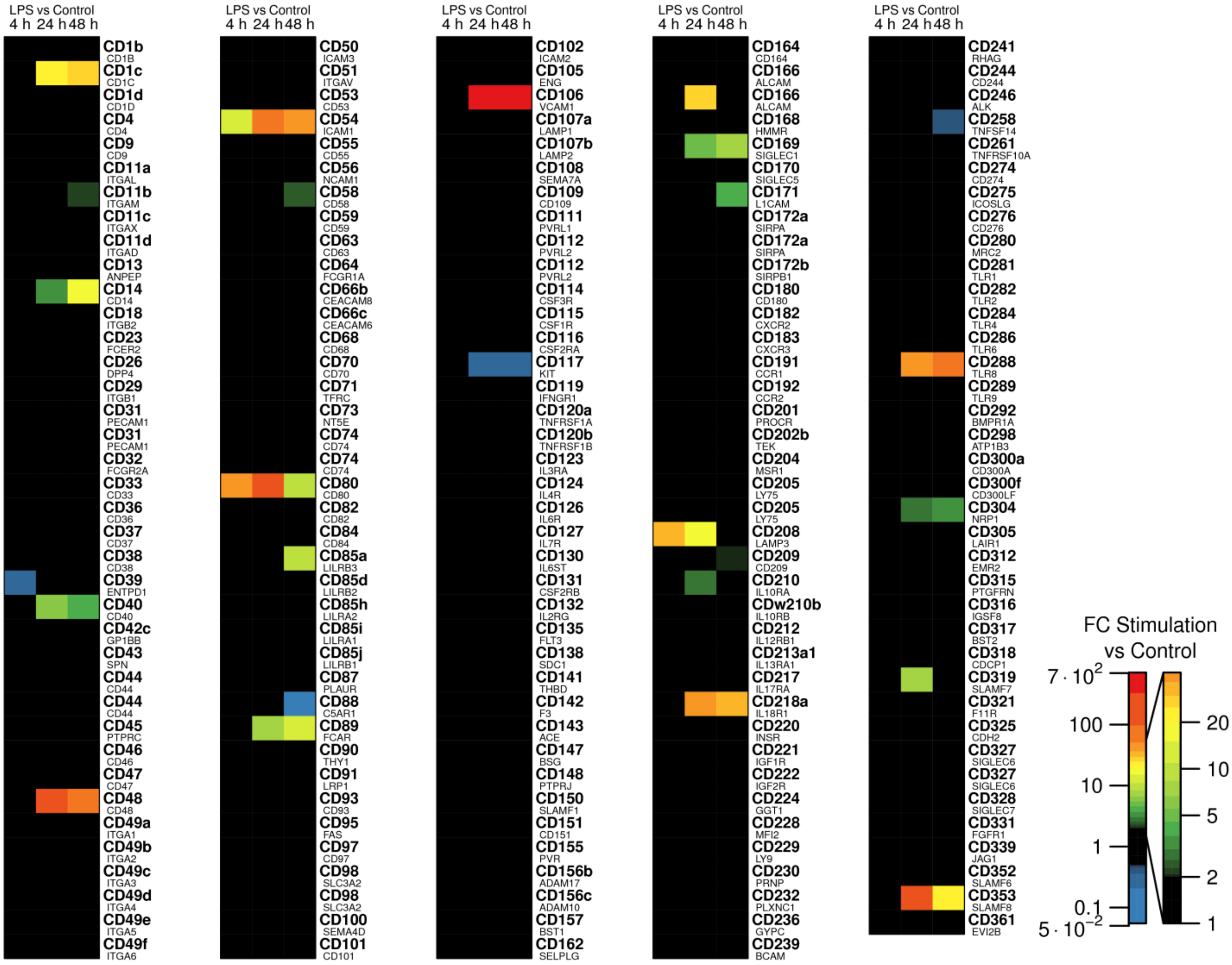

Supplementary Figure S3. CD antigens identified and regulated in THP-1 cells. Overview of the 180 identified glycoproteins representing CD antigens identified in the LPS time course in THP-1 cells at time points 4h, 24h and 48h. The color denotes significant up- or downregulation by LPS treatment according to the legend.

Supplementary Figure S4

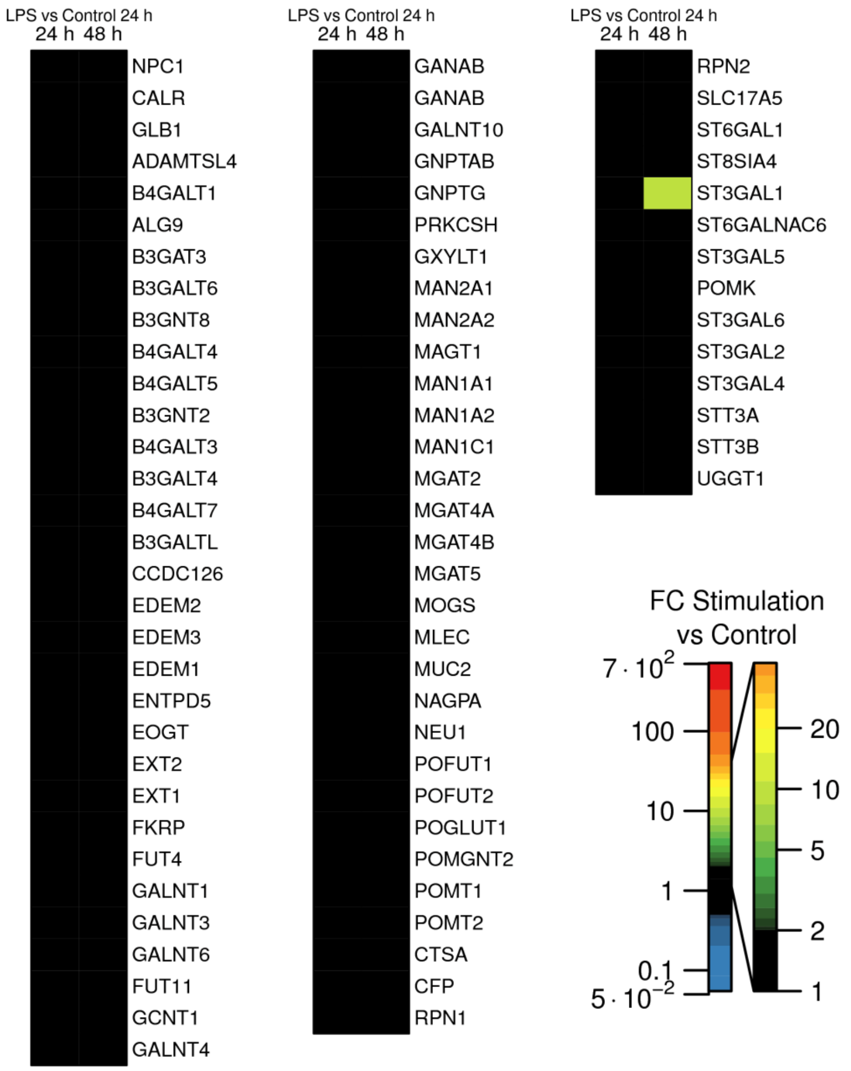

Supplementary Figure S4. Overview of the 76 glycoproteins identified in the LPS time course in CD14+ monocytes annotated as "protein glycosylation" (GO: 0006486) at time points 24h, or 48h. The color code denotes significant up- or downregulation by LPS treatment according to the legend.

Supplementary Figure S5

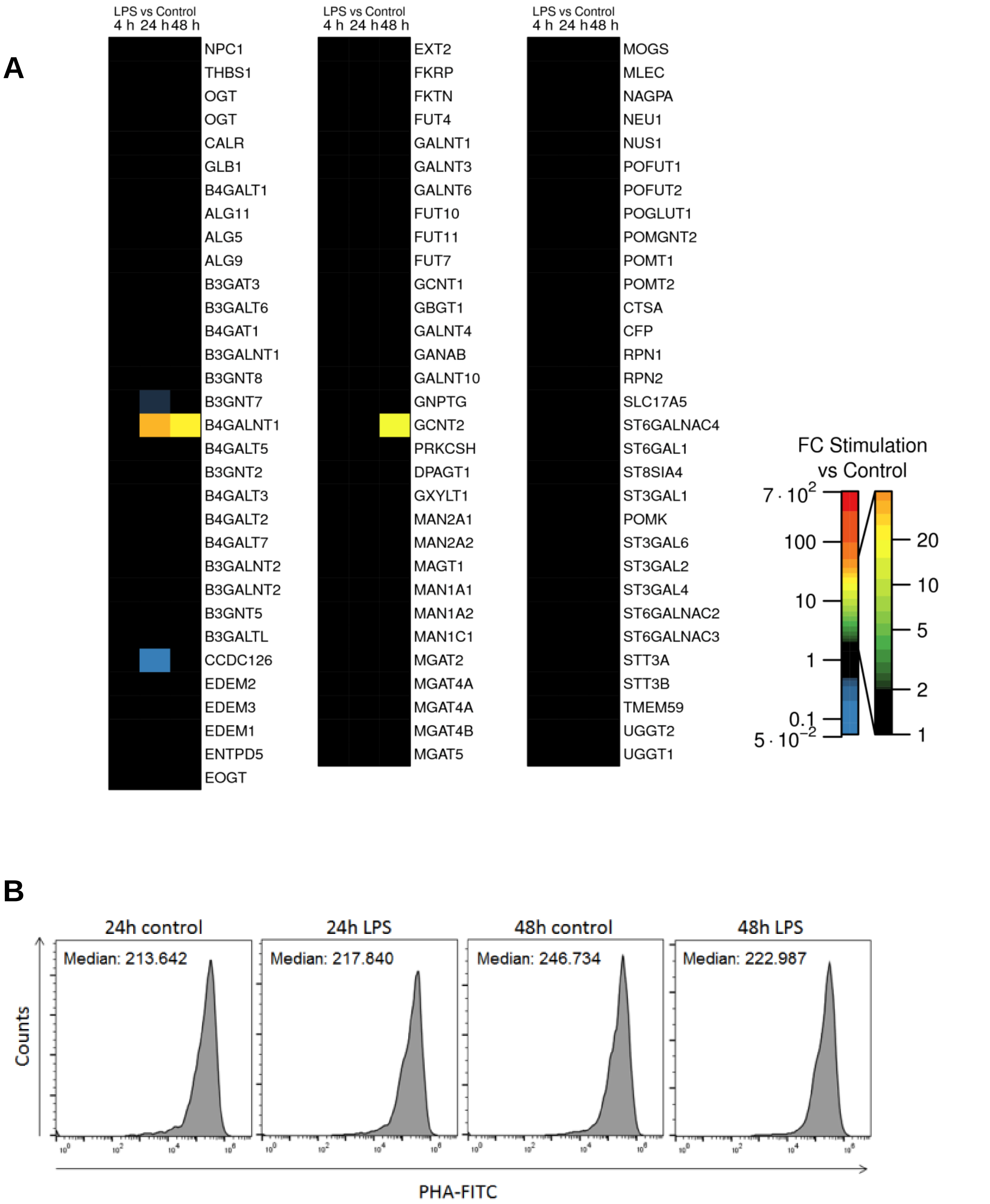

Supplementary Figure S5. (A) Overview of the 92 glycoproteins identified in the LPS time course in THP-1 cells annotated as “protein glycosylation” (GO: 0006486) at time points 4 h, 24 h, or 48 h. The color code denotes significant up- or downregulation by LPS treatment according to the legend. (B) Branching degree of THP-1 cell surface glycans. Flow cytometry histogram plots of live, unfixed THP-1 cells at t(0) (control t(0)) or after 24 h and 48 h of incubation (control) or stimulation with LPS (LPS) stained with PHA-L-Alexa488 lectin. No changes in branched complex glycoconjugates were detected.

Supplementary Figure 6

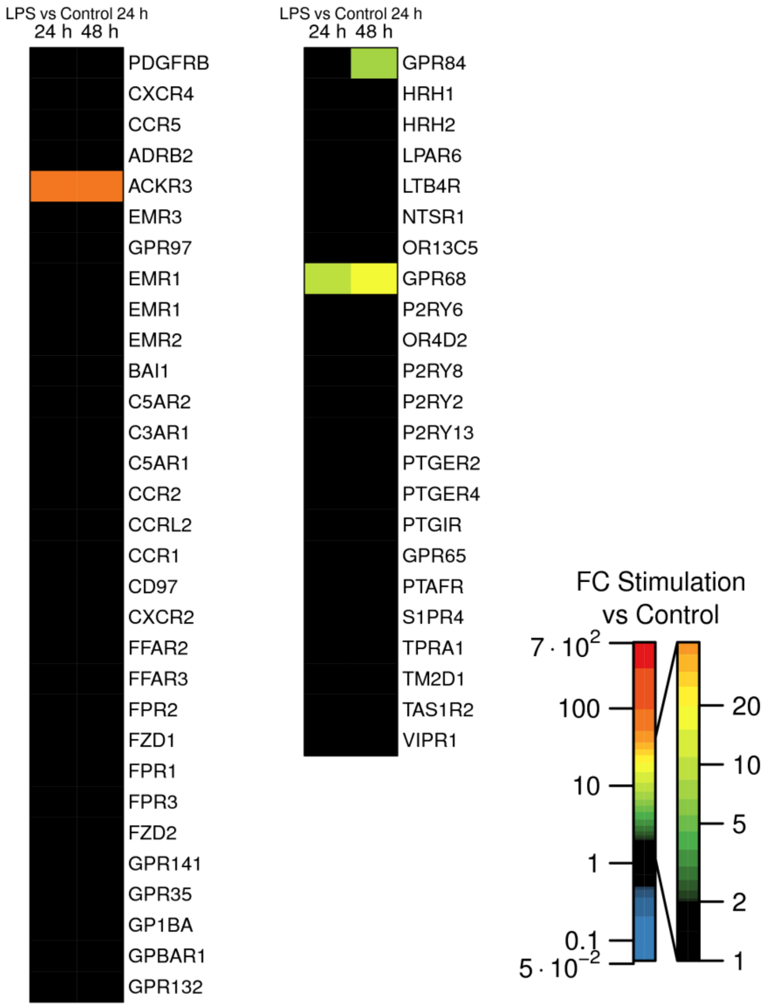

Supplementary Figure S6. Overview of the 52 identified glycoproteins with G protein-coupled activity (GO:0004930) identified in the LPS time course in CD14$^+$ monocytes at time points 24h and 48h. The color denotes significant up- or downregulation by LPS treatment according to the legend.

Supplementary Figure 7

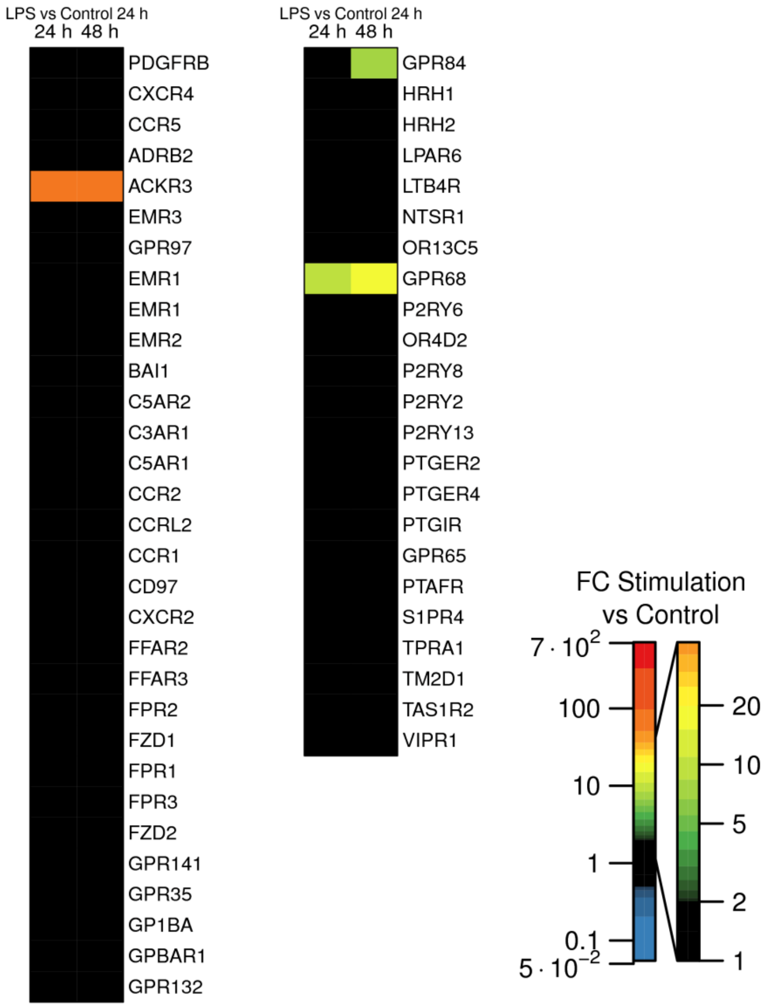

Supplementary Figure S7. Overview of the 52 identified glycoproteins with G protein-coupled activity (GO:0004930) identified in the LPS time course in CD14$^+$ monocytes at time points 24h and 48h. The color denotes significant up- or downregulation by LPS treatment according to the legend.

Supplementary Figure S8

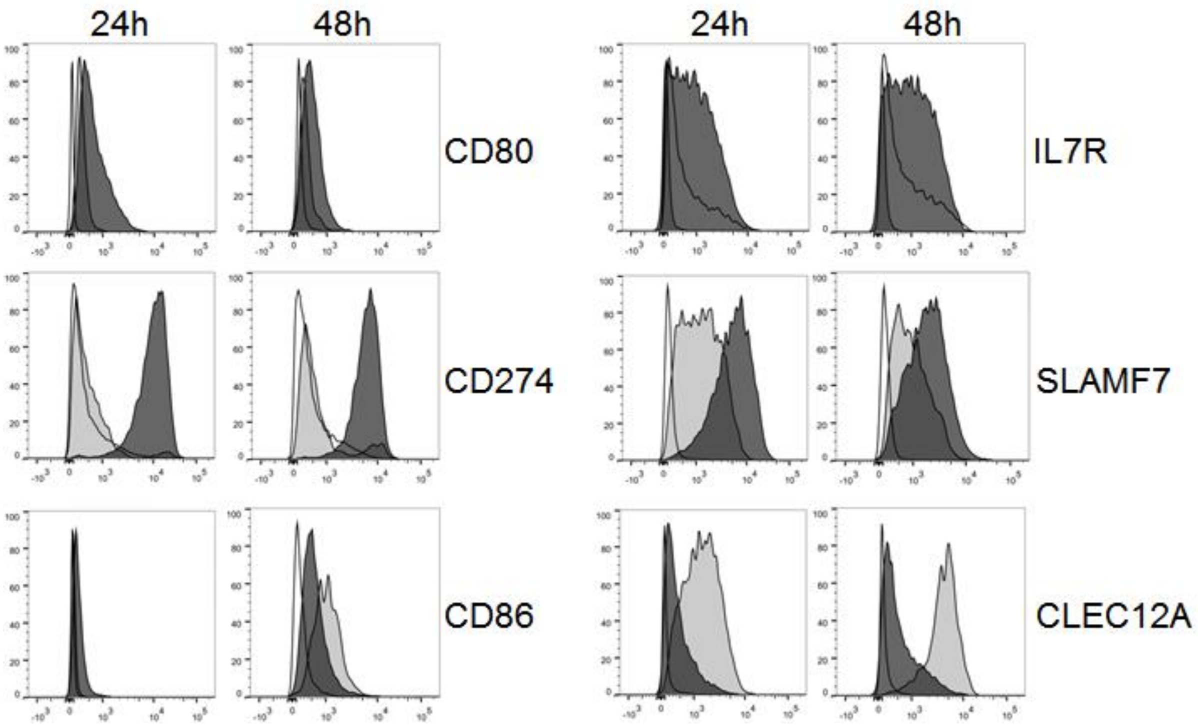

Supplementary Fig. S8. Verification of proteomic results by flow cytometry ($CD14^+$ monocytes). Cell surface expression of CD80, IL7R, CD274, SLAMF7, CD86, and CLEC12A on $CD14^+$ monocytes isolated by MACS and stimulated with or without 50 ng/ml LPS for 24h or 48h. Light grey: expression at the indicated time point of unstimulated controls, dark grey: expression at the indicated time points after LPS treatment, white: isotype control antibodies. The data shown are representative of two different donors independently analyzed.

Supplementary Table S1

Primer List

PPIB	Fw: ATGTAGGCCGGGTGATCTTT	Rv: TGAAGTTCTCATCGGGGAAG
HPRT1	Fw: GACCAGTCAACAGGGGACAT	Rv: AACACTTCGTGGGGTCCTTTTC
TNFα	Fw: TTCTCCTTCCTGATCGTGGC	Rv: ACTCGGGGTTCGAGAAGATG
MMP9	Fw: CAACTACGACACCGACGAC	Rv: TGGCCTTGGAAGATGAATGGA
IL4I1	Fw: CACTCGCCCGAAGACATCTA	Rv: AGAGCGTGTGCCTTTCAAAC
STEAP4	Fw: TCAGCCTGGCTCAGTGATTC	Rv: TCGGAACTCTCTCCAGTTGAC
EBI3	Fw: CCCTACGTGCTCAATGTCAC	Rv: CCCTGACGCTTGTAACGGA
GPR84	Fw: TGATCTCCTCTACTGCACGC	Rv: AAGGAGGAGCCCAAATACCC
LAMP3	Fw: AGTGGGAGCCTATTTGACCG	Rv: TGGAGGCTCTGTTCACTCAC
DPEP2	Fw: AAGGGCGTCCACTCCTTCTA	Rv: ACAGCATCTGAGACATGGGA
ITGB8	Fw: GTCCAGAATGTGGATGGTGTG	Rv: ACTGAGCAGCCTTTGCTTATTAAA

Supplementary Table S2: LPS regulated glycoproteins with significant FC (FC log(2)>2, $p_{adj.}$<0.05) in the glycoproteomics data set of monocytes compared to the FC determined in the THP-1 data set at 24h and 48h[§]

			Log(2) FC monocytes		Log(2) FC THP-1	
			LPS vs contr 24h		LPS vs contr	
**Gene name***	**Accession**	**Protein name**	24h	48h	24h	48h
Upregulated						
**CD274**	Q9NZQ7	Programmed cell death 1 ligand 1	11.2	10.9	1.7	1.8
**IL7R**	P16871	Interleukin-7 receptor subunit alpha	8.1	6.2	1.4	0.4
**CA12**	O43570	Carbonic anhydrase 12	8.0	7.6	nd	nd
ITGB8	P26012	Integrin beta-8	7.7	4.0[$]	nd	nd
SLAMF1	Q13291	Signaling lymphocytic activation molecule	7.6	5.4[$]	1.0	0.0
**LAMP3**	Q9UQV4	Lysosome-associated membrane glycoprotein 3	6.9	6.7	4.3	3.0
FAM20A	Q96MK3	Protein FAM20A	6.5	5.4[$]	nd	nd
**KREMEN1**	Q96MU8	Kremen protein 1	6.1	5.6	nd	nd
**PTGS2**	P35354	Prostaglandin G/H synthase 2	5.9	3.3	3.2	0.8
**GGT5**	P36269	Gamma-glutamyltransferase 5	5.7	4.3	nd	nd
**SLCO4A1**	Q96BD0	Solute carrier organic anion transporter family member 4A1	5.6	4.7	1.5	-0.1
**ACKR3**	P25106	Atypical chemokine receptor 3	5.5	5.9	nd	nd
**CD80**	P33681	T-lymphocyte activation antigen CD80	5.4	5.7	6.0	3.6
DNER	Q8NFT8	Delta and Notch-like epidermal growth factor-related receptor	5.3	2.4[$]	nd	nd
**CD276**	Q5ZPR3	CD276 antigen	4.9	4.4	0.2	-0.1
**MARCO**	Q9UEW3	Macrophage receptor MARCO	4.1	3.6	nd	nd
SIGLEC10	Q96LC7	Sialic acid-binding Ig-like lectin 10	4.0	2.3[$]	6.0	-0.9
**NT5E**	P21589	5-nucleotidase	4.0	3.8	-0.2	-0.1
**PROCR**	Q9UNN8	Endothelial protein C receptor	3.8	2.9	0.5	1.1
**PLAU**	P00749	Urokinase-type plasminogen activator	3.4	2.7	0.7	0.9
**GPR68**	Q15743	Ovarian cancer G-protein coupled receptor 1	3.2	3.8	nd	nd
**TPST1**	O60507	Protein-tyrosine sulfotransferase 1	3.2	3.2	1.5	0.1
**TMEM132A**	Q24JP5	Transmembrane protein 132A	3.1	2.6	nd	nd
SLC7A5	Q01650	Large neutral amino acids transporter small subunit 1	2.9	1.9[$]	0.3	0.7
**ACVRL1**	P37023	Serine/threonine-protein kinase receptor R3	2.8	2.6	0.2	1.5
CHST2	Q9Y4C5	Carbohydrate sulfotransferase 2	2.8	0.2[$]	nd	nd
**SLC43A3**	Q8NBI5	Solute carrier family 43 member 3	2.6	2.1	0.1	0.2
ST3GAL1	Q11201	CMP-N-acetylneuraminate-beta-galactosamide-alpha-2,3-sialyltransferase 1	2.5[$]	2.9	0.2	0.3
**ABCA1**	O95477	ATP-binding cassette sub-family A member 1	2.5	2.1	nd	nd
**ITGAV**	P06756	Integrin alpha-V	2.4	2.0	0.0	-0.1
SEMA4A	Q9H3S1	Semaphorin-4A	2.2[$]	2.5	0.3	0.4
GPR84	Q9NQS5	G-protein coupled receptor 84	2.0[$]	2.8	4.9	3.8
ADAMDEC1	O15204	ADAM DEC1	2.0[$]	3.1	5.4	4.9
CD82	P27701	CD82 antigen	2.0[$]	2.1	0.2	0.2
ICAM1	P05362	Intercellular adhesion molecule 1	1.8[$]	2.3	5.4	5.3
MPZL1	O95297	Myelin protein zero-like protein 1	1.3[$]	2.5	1.3	1.6

Supplementary Table S2 continued

Downregulated						
MR1	Q95460	Major histocompatibility complex class I-related gene protein	-0.2$	-2.1	0.3	0.2
HLA-DMA	P28067	HLA class II histocompatibility antigen, DM alpha chain	-1.1$	-2.6	nd	nd
MGAM	O43451	Maltase-glucoamylase, intestinal	-1.2$	-2.3	0.1	0.3
SLC23A2	Q9UGH3	Solute carrier family 23 member 2	-1.3$	-2.5	0.8	0.2
TLR7	Q9NYK1	Toll-like receptor 7	-1.7$	-3.9	4.8	5.5
CD244	Q9BZW8	Natural killer cell receptor 2B4	-1.7$	-2.5	0.1	0.3
HLA-DPA1	P20036	HLA class II histocompatibility antigen, DP alpha 1 chain	-1.9$	-2.8	1.7	2.6
**FGG**	P02679	Fibrinogen gamma chain	-1.9	-2.4	nd	nd
MPEG1	Q2M385	Macrophage-expressed gene 1 protein	-1.9$	-2.9	1.2	1.1
PTGFRN	Q9P2B2	Prostaglandin F2 receptor negative regulator	-2.0	-0.2$	-0.3	-0.2
**CD36**	P16671	Platelet glycoprotein 4	-2.1	-2.2	0.5	0.5
FN1	P02751	Fibronectin	-2.1$	-1.5$	1.0	-0.1
HLA-DMB	P28068	HLA class II histocompatibility antigen, DM beta chain	-2.1$	-3.1	nd	nd
FUCA1	P04066	Tissue alpha-L-fucosidase	-2.1$	-2.6	0.0	-0.3
CFH	P08603	Complement factor H	-2.1	-0.8$	nd	nd
**FGB**	P02675	Fibrinogen beta chain	-2.2	-3.6	nd	nd
HLA-DPB1	P04440	HLA class II histocompatibility antigen, DP beta 1 chain	-2.3$	-4.6	nd	nd
**HAVCR2**	Q8TDQ0	Hepatitis A virus cellular receptor 2	-2.3	-1.9	nd	nd
C1R	P00736	Complement C1r subcomponent	-2.3	-0.7$	nd	nd
**FGL2**	Q14314	Fibroleukin	-2.5	-2.8	0.7	-1.4
A2M	P01023	Alpha-2-macroglobulin	-2.8	-0.7$	-0.5	0.3
**MSR1**	P21757	Macrophage scavenger receptor types I and II	-3.1	-3.6	-1.0	-1.2
CD9	P21926	CD9 antigen	-3.4	-1.3$	4.8	2.3
SLCO2B1	O94956	Solute carrier organic anion transporter family member 2B1	-3.4	-2.8$	nd	nd
**GPNMB**	Q14956	Transmembrane glycoprotein NMB	-3.4	-3.1	0.7	0.7
NRG1	Q02297	Neuregulin-1	-3.9	-1.8$	nd	nd
FOXRED2	Q8IWF2	FAD-dependent oxidoreductase domain-containing protein 2	-4.6	-2.0$	-0.1	0.1
COLEC12	Q5KU26	Collectin-12	-5.6	-4.6$	nd	nd
**DPEP2**	Q9H4A9	Dipeptidase 2	-5.7	-3.3	nd	nd

$Only monocyte glycoproteins with a log(2) FC > ±2 at a single time point or both time points are presented.
*In bold: glycoproteins with statistical significant fold change at both time points 24h and 48h.
$Not statistically significant at this time point. nd = not detected.
